# Targeting CDK1 in cancer: mechanisms and implications

**DOI:** 10.1038/s41698-023-00407-7

**Published:** 2023-06-13

**Authors:** Qiushi Wang, Ann M. Bode, Tianshun Zhang

**Affiliations:** The Hormel Institute, University of Minnesota, 801 16th Ave NE, Austin, MN 55912 USA

**Keywords:** Targeted therapies, Chemotherapy

## Abstract

Cyclin dependent kinases (CDKs) are serine/threonine kinases that are proposed as promising candidate targets for cancer treatment. These proteins complexed with cyclins play a critical role in cell cycle progression. Most CDKs demonstrate substantially higher expression in cancer tissues compared with normal tissues and, according to the TCGA database, correlate with survival rate in multiple cancer types. Deregulation of CDK1 has been shown to be closely associated with tumorigenesis. CDK1 activation plays a critical role in a wide range of cancer types; and CDK1 phosphorylation of its many substrates greatly influences their function in tumorigenesis. Enrichment of CDK1 interacting proteins with Kyoto Encyclopedia of Genes and Genomes (KEGG) pathway analysis was conducted to demonstrate that the associated proteins participate in multiple oncogenic pathways. This abundance of evidence clearly supports CDK1 as a promising target for cancer therapy. A number of small molecules targeting CDK1 or multiple CDKs have been developed and evaluated in preclinical studies. Notably, some of these small molecules have also been subjected to human clinical trials. This review evaluates the mechanisms and implications of targeting CDK1 in tumorigenesis and cancer therapy.

## Introduction

Cyclin dependent kinases (CDKs) are serine/threonine kinases that form a complex with cyclin proteins, a process that is essential for full activation of their kinase activity. CDKs play critical roles in the control of cell division and modulation of transcription in response to extracellular and intracellular stimuli^1^. CDKs are involved in many crucial processes and are associated with several disease conditions, such as Alzheimer’s disease^2^, Parkinson’s disease^3^, stroke^4^, HIV^5^, and cancer^6,7^. The CDK protein family comprises twenty kinases (CDK1-20). CDK1-6 and CDK14-18 are involved in cell cycle and CDK7-13 and CDK19-20 are associated with the function of transcription in gene control^8,9^. CDK1 is the only CDK in mammals that is essential for cell cycle progression^10^. It promotes the G2/M and G1/S transitions, as well as G1 progression^11^. Unrestricted cell proliferation, an indicator of malignancy, is normally driven by alterations in CDK1 activity. The expression of CDKs fluctuates cyclically throughout the cell cycle^12^. Cancer is a disease of abnormal cell proliferation and occurs when cells evade normal growth or division restrictions. Oncogenic transformation often entails derangement of the mechanisms that ensure the stable inheritance of genes and chromosomes during mitotic cell division^13^. CDKs play important roles in both the commitment to cell division and the quality control mechanisms that safeguard genome integrity. They represent obvious, but potentially risky, therapeutic targets in treating human cancers^14^. In addition to presenting the frequency of overexpression in different cancer types, CDKs have been shown to function as oncogenes or were identified as frequently overexpressed secondary oncogenes in several types of cancer, including melanoma and lung cancer. In these cancers, CDKs are not the primary drivers of cancer but are overexpressed in conjunction with other oncogenes^15–17^. For instance, CDKs are highly expressed in non-small cell lung cancer with EGFR mutations^16^. CDK/cyclin activity is mediated by physiological CDK inhibitors or CKIs. Over the last decade, substantial progress has been made in discovering and developing novel small molecule CKIs^18–22^. This area of drug discovery has adopted novel research strategies that are different from the classic reversible ATP competitive or non-competitive action modes. Traditional kinase inhibitory molecules include irreversible ATP competitive drugs, reversible and irreversible structural inhibitors, CDK degrading drugs, and inhibitory CDK binding antibodies. Newer drugs have opened an avenue to interrogate, for example, new and more challenging transcriptional CDK targets^22^. A number of selective inhibitors or pan-inhibitors of CDK1 have been produced over past decades. Inhibition of the expression and activation of CDK1 effectively suppresses oncogenic cell function in many cancer types. Notably, some small molecules targeting CDK1 have already been studied in clinical trials. In this review, we evaluate the critical role and mechanisms of CDK1 in tumorigenesis. Additionally, we examine the current CDK1 inhibitors that have been evaluated in preclinical and clinical studies for cancer therapy.

## CDK expression in cancer

**Fig. 1:**
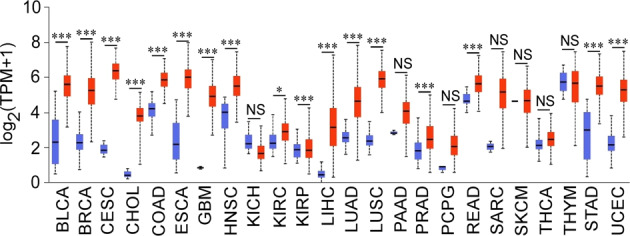
Expression levels of CDK1 in various cancers. Comparison of the expression of CDK1 between tumor (red) and normal (blue) tissues. For the boxplots, the center line of the box indicates the median. The upper boundary of the box represents the upper quantile, while the bottom boundary of the box represents the lower quartile. The top and bottom ends of the whiskers indicate the maximum and minimum values, respectively. (*p < 0.05, **p < 0.01, ***p < 0.001, NS: no significant difference).

According to The Cancer Genome Atlas (TCGA) UALCAN database^23,24^, CDKs are significantly upregulated in many cancerous tissues compared to normal tissues, indicating a widespread increase in their expression. (Fig. 1, Supplementary Table 1, Supplementary Fig. 1). Based on these results, CDK1, CDK2, CDK4, CDK5, and CDK7 are the top 5 CDKs that are highly expressed in cancer tissues compared to normal tissues. Overall, compared with normal tissues, the expression of CDK4 and CDK5 are higher in 18 out of 24 or 75% of the cancers listed. CDK1 is significantly higher in 17 out of 24 or 70.8% of the cancers listed and CDK2 and CDK7 are higher in 16 out of 24 or 67% of the cancers listed.

**Fig. 2:**
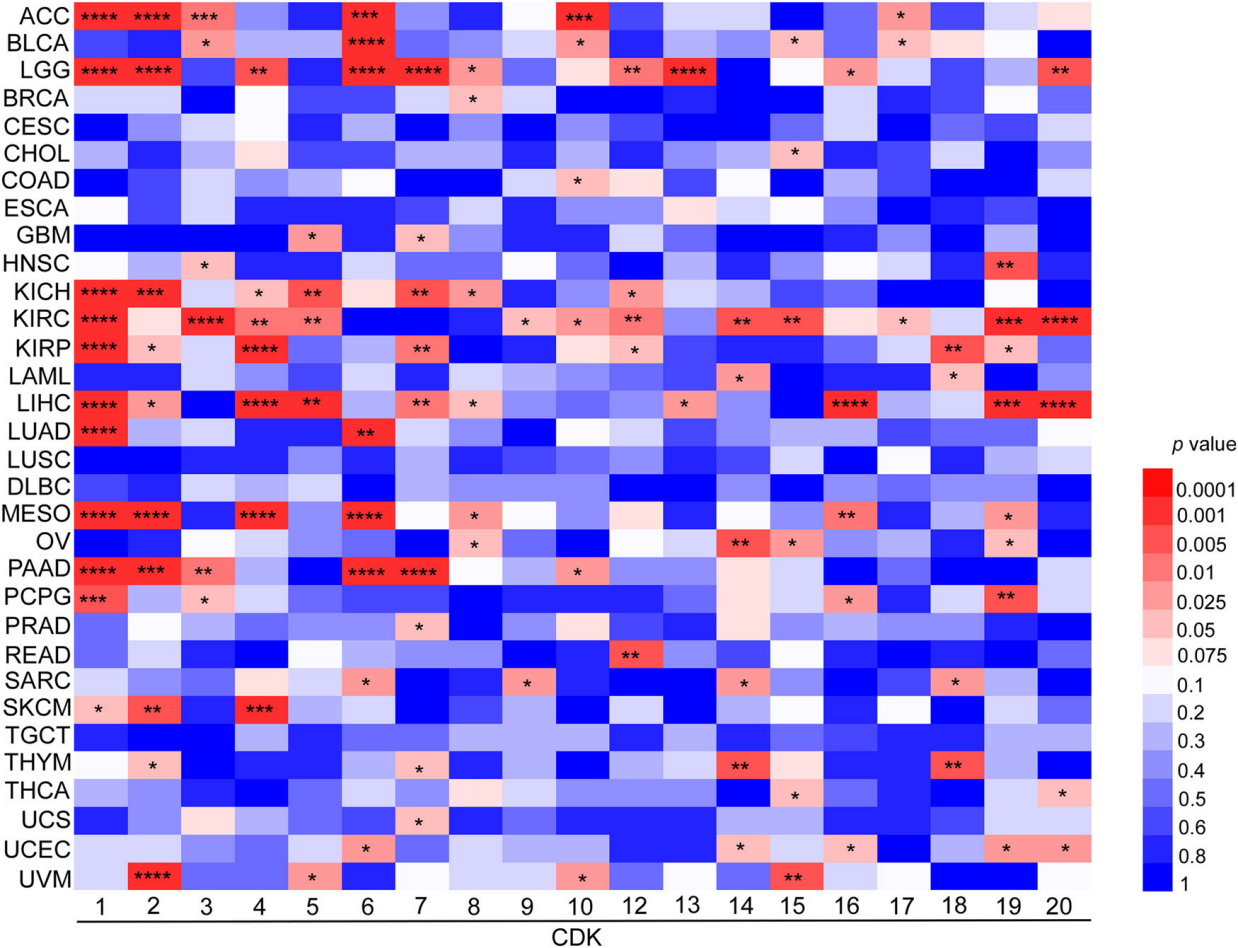
Correlation between CDK expression and patient overall survival. The survival data derived from the ULCAN database are categorized into two groups for analysis. Groups include high CDKs expression (values above upper quartile) and low/medium CDKs expression (values below upper quartile). The differences (*p* value) between groups are demonstrated by heatmap (*p < 0.05, **p < 0.01, ***p < 0.001, ****p < 0.0001).

In addition, information from the database indicates that high expression of CDKs is closely correlated with the overall survival rate in 32 different cancer types (Supplementary Table 1, Fig. 2). Overall, the data indicate that the top 5 CDKs, CDK1 (11/32 or 34.4%), CDK2 (10 /32 or 31.3%), CDK6 (8/32 or 25%), CDK7 (9/32 or 28.1%), and CDK19 (8/32 or 25%), are closely associated with survival probability in various cancers.

CDKs are highly expressed in cancer tissues and closely associated with survival probability in multiple cancer types. Collectively, these results indicate that targeting CDKs, and especially CDK1, could be a critical strategy for cancer treatment. The remainder of this review focuses on mediators, substrates, and inhibitors of CDK1 in cancer.

## Upstream mediators of CDK1

The upstream modulators of CDK1 (Supplementary Table 2) in cancer include various molecular factors that can positively or negatively influence CDK1 activation, amplification, transcription, and expression.

### Positive upstream modulators of CDK1

#### Activators of CDK1

CDK1 is essential for cell division during mitosis. It helps form the spindle and aligns chromosomes by recruiting and activating key proteins involved in kinetochore formation. CDK1 activity ensures proper chromosome orientation and segregation and is critical for the successful assembly of the mitotic apparatus and chromosome alignment. The activation of CDK1 requires phosphorylation on Thr161 or dephosphorylation on Thr14 and Tyr15^25,26^. CDK7 mediates G1 cell cycle arrest and extrinsic apoptosis by increasing phosphorylation of CDK1 at Thr161^27^. Protein tyrosine phosphatase receptor type F (LAR) also increases focal adhesion by enhancing CDK1 activation at Thr161^28^. Nucleolar protein 11 (NOL11) and CDK5 regulatory subunit associated protein 3 (C53) delay cell entry into mitotic phase though dephosphorylation of CDK1 on Tyr15^29,30^. Cell division cycle 25 (CDC25) is a dual-specificity phosphatase, which counteracts G2/M checkpoint activation by removing inhibitory phosphate groups (Thr14 or Tyr15) from CDK1 and are themselves negatively modulated by checkpoint kinase 1 (CHK1)^31^. CDC25 proteins include CDC25A, CDC 25B, and CDC 25C. They mediate meiosis through activation of CDK1 by dephosphorylation on Thr14 and Tyr15^27,32–35^. Associated with CDC25 mediation of activation of CDK1 are several molecules, including CDK2, beclin 1 (BECN1), tetramerization domain containing 12 (KCTD12), nucleophosmin (NPM), and minichromosome maintenance 10 replication initiation factor (MCM10), each of which facilitates activation of CDK1 by mediating CDC25 activity^36–43^. For example, BECN1 translocases into the nucleus, where it interacts with CDC25C and CHK2, resulting in promotion of radiation-induced G2/M arrest through promotion of CDK1 activity^40^. Additionally, other molecules, such as Aurora A kinase (AURKA)^44^, 6-phosphofructo-2-kinase (PFKFB3)^45^, ubiquitin C-terminal hydrolase L1 (UCH-L1)^46^, microtubule-associated serine/threonine kinase-like (MASTL)^47^, testis-specific protein Y-encoded (TSPY)^48^, karyopherin subunit beta 1 (KPNB1)^49^, and STIL centriolar assembly protein (**SIL**), all promote CDK1 activity (Supplementary Table 2)^50^.

#### Transcriptional modulation of CDK1 and upregulation of CDK1 expression (Supplementary Table 2)

E2F transcription factor (E2F)-dependent transcription controls both G1/S- and G2/M-associated genes. Specifically, E2F1, E2F2, and E2F3 enhance CDK1 transcription by binding to the positive-acting E2F site in the CDK1 promoter, which results in increased CDK1 expression^51^. Mortality factor 4 like 1 (MRG15), a chromatin modulator, is a highly conserved protein present in complexes containing histone acetyltransferases (HATs), as well as histone deacetylases (HDACs). MRG15 acts in the HAT complex through its acetylation of histone H4 at the CDK1 promoter to activate transcription^52^. The cysteine-rich CXC domain of Lin-54 DREAM MuvB core complex component (LIN54) is a novel DNA-binding domain that binds to the CDK1 promoter in a sequence-specific manner^53^. Besides directly binding with the CDK1 promoter, several molecules also modulate CDK1 transcriptional activation. For example, CDK1 is a direct transcriptional target of centromere-associated protein E (CENPE) in primary pulmonary artery smooth muscle cells. The overexpression of CENPE significantly increases CDK1 promoter activity, whereas the deletion of CENPE markedly decreases promotor activity^54^, which attenuates CDK expression. Sp1 transcription factor (SP1), initially identified as a transcription factor, plays a crucial role in normal biological processes, neoplastic development, and tumor migration^55^. Dual-luciferase reporter assay results showed the direct effect of SP1 on the transcriptional activation of CDK1^56^. Knockdown of ribosomal protein S9 (RPS9) inhibits the growth of human colon cancer cells at the G2/M phase by downregulating CDK1 expression at the promoter level^57^.

Several molecules enhance tumor cell growth, migration, or invasion by upregulating the expression of CDK1 in different ways. Among them, chondroitin polymerizing factor (CHPF), co-stimulatory molecule (CD276), and papillomavirus E6 (E6) enhance CDK1 expression by increasing the expression of transcription factor E2F1. Knocking down expression of CHPF or CD276 maintains proliferation or modulates differentiation by mediating E2F1/CDK1 expression in malignant melanoma and endothelial progenitor cells, respectively^58,59^. NOP2/sun RNA methyltransferase 2 (NSUN2) and death-associated protein 5 (DAP5) promote CDK1 expression by enhancing CDK1 translation^60–62^. NSUN2 methylates *CDK1* mRNA in vitro and in cells, and that methylation by NSUN2 enhances CDK1 translation influencing cell growth and survival during mitosis^60,61^. Oncogenic action of RNA binding motif protein 7 (RBM7) and histone deacetylase 6 (HDAC3) controls cell progression by stabilizing *CDK1* mRNA and protein levels, respectively. RBM7 directly binds to the AU-rich elements (AREs) in the 3’-UTR of *CDK1* mRNA, which contributes to the stability of *CDK1* mRNA by lengthening CDK1 half-life in breast cancer^63^. HDAC3 mediates G2/M phase progression mainly through post translational stabilization of the CDK1 protein by controlling CDK1 ubiquitination^64^. Somatic mutations in DNA methyltransferase 3 alpha (DNMT3A) have been identified in approximately 25% of patients with LAML *DNMT3A* mutation that occurs in the early stages of LAML and is regarded as a pre-leukemic gene mutation^65^. *DNMT3A* mutation can induce CDK1 overexpression and promote leukemogenesis^66^. Additionally, several other molecules also mediate cell proliferation, metastasis, or survival by enhancing CDK1 expression (Supplementary Table 2).

### Negative upstream modulators of CDK1

#### Mediators that decrease activation of CDK1

Many molecules markedly upregulate the activity of CDK1 in tumorigenesis, whereas negative upstream mediators of CDK1 also widely exist. These negative modulators are usually tumor suppressors that inhibit CDK1 activation in tumor progression. As indicated earlier, dephosphorylation on Thr14 and Tyr15 or phosphorylation at Thr161 is required for the full activation of CDK1^25^. Wee1-like protein kinase 1 (WEE1)^67–69^ and membrane associated tyrosine/threonine 1 (MYT1) kinase^70–72^ inhibit CDK1 activation by phosphorylation at Thr14 and Tyr15, and this modification plays a crucial role in the G2–M cell-cycle checkpoint arrest for DNA repair before mitotic entry^73^. Besides these two important kinases, many other key molecules also inhibit CDK1 activation by phosphorylation of Thr14 and Tyr15 or dephosphorylation of Thr161 (Supplementary Table 2). For example, phosphatase and tensin homolog (PTEN) is one of the most important and well-studied tumor suppressor proteins. Downregulation of PTEN by siRNA in cells increases phospho-WEE1 (Ser642), but decreases phospho-CDK1 (Tyr15), resulting in decreased G2/M cell cycle arrest^74^. Dual specificity tyrosine phosphorylation regulated kinase 1A (DYRK1A) demonstrates its tumor suppressive function by mediating phosphorylation of Tyr15 and Thr161 in glioblastoma cells^75^. CDC25 is known to activate CDK1 by dephosphorylating residues Thr14 and Tyr15^31^. Checkpoint kinase 1 (CHEK1), one of the critical transducers in DNA damage/replication checkpoints, prevents entry into mitosis through its inhibition of CDC25 and CDK1 activity^76,77^. Fibroblast growth factor 1 (FGF1) also causes dephosphorylation of the CDC25C phosphatase inducing inactivation of the cyclin B1/CDK1 complex. Kinesin family member 22 (KIF22) is a microtubule-dependent molecular motor protein with DNA-binding capacity. CDC25C is a direct transcriptional target of KIF22 and inhibition of KIF22 increases CDC25C expression and cyclin-dependent kinase 1 (CDK1) activity, resulting in delayed mitotic exit^78^. Other proteins can also affect CDK1 activity but with no effect on phosphorylation of Thr14 and Tyr15 or dephosphorylation of Thr161. For example, death effector domain containing (DEDD) protein participates in apoptosis signaling, which inhibits activation of CDK1 but does not affect the phosphorylation status at Thr14, Tyr15, or Thr161^79,80^. Apart from these proteins, several other molecules can also inhibit CDK1 activation (Supplementary Table 2).

#### Mediators that decrease CDK1 expression and nuclear translocation

In addition to CDK1 activation, CDK1 expression is also tightly modulated. Eukaryotic cells utilize two major routes to effectively target a wide range of proteins for degradation, including the ubiquitin/proteasome system and the autophagy/lysosome pathway^81^. Double-stranded RNA-activated protein kinase (PKR) is a serine/threonine interferon (IFN)-inducible kinase that plays an important role in the regulation of gene expression at both transcriptional and translational levels. PKR-mediated Tyr4-phosphorylation facilitates CDK1 ubiquitination and proteasomal degradation^82^. CDK1 accumulation in patients’ tumors shows a negative correlation with beta-transducing repeat containing E3 ubiquitin protein ligase (BTRC) and exhibits a positive correlation with the degree of tumor malignancy. BTRC controls the lysosome-mediated degradation of CDK1, the accumulation of which correlates with tumor malignancy^83^. Histone deacetylase 6 (HDAC6) plays a dual role in the autophagy/lysosome pathway. It controls the fusion of autophagosomes to lysosomes by promoting F-actin remodeling in a cortactin-dependent manner^84^. In contrast, upon proteasome inhibition, HDAC6 is recruited and relocates to polyubiquitin-positive aggresomes^85^. Ubiquitin-binding protein P62 (P62) is a key protein in the autophagic clearance of polyubiquitinated proteins^86^. CDK1 degradation reportedly involves p62/HDAC6-mediated selective autophagy^87^. Additionally, the TNF-like WEAK inducer of apoptosis (TWEAK)^88^, human enhancer of invasion, clone 10 (HEI10)^89^, and sialophorin (SPN)^90^ also mediate CDK1 expression by inducing CDK1 degradation, inhibiting CDK1 expression or nuclear translocation (Supplementary Table 2).

## Downstream substrates of CDK1

As a serine/threonine protein kinase, CDK1 is reported to phosphorylate a number of substrates, including both tumor promotors and tumor suppressors (Supplementary Table 3).

### CDK1 tumor promotor substrates

Increasing evidence suggests that CDK1 phosphorylates downstream substrates that play critical roles in cancer progression signaling pathways. The B-Raf proto-oncogene, serine/threonine kinase (BRAF), as a critical activator of the mitogen-activated protein kinase (MAPK) cascade during mitosis. CDK1/cyclin B directly phosphorylates BRAF at Ser144, which is required for mitotic activation and subsequent activation of the MAPK cascade^91^. Extracellular signal regulated kinase 3 (ERK3) is an atypical MAPK that is suggested to play a role in cell cycle progression and cellular differentiation. CDK1 can also phosphorylate ERK3 at Thr698, which acts in a cell-cycle-dependent manner^92^. Androgen receptor (AR) is the principal molecule in prostate cancer etiology and therapy and its re-activation remains a major challenge during treatment of prostate tumors that relapse after castration therapies. CDK1 phosphorylates the AR at Ser81 or Ser515 promoting prostate tumor progression^93–95^. Hypoxia-inducible factor 1α (HIF1A) is a major mediator of tumor physiology, and its activation is correlated with tumor progression, metastasis, and therapeutic resistance^96,97^. CDK1 stabilizes HIF1A through direct phosphorylation of Ser668 to promote tumor growth^98^. YAP is a downstream effector of the Hippo pathway of cell-cycle control that plays important roles in tumorigenesis. CDK1 phosphorylates YAP promoting mitotic defects and cell motility and is essential for neoplastic transformation^99^. TAZ is also a downstream effector of the Hippo pathway, which plays important roles in cancer and stem cell biology. CDK1 phosphorylation of TAZ in mitosis inhibits its oncogenic activity^100^. Additionally, the adaptor protein, ajuba LIM protein (AJUBA), is a positive mediator of YAP oncogenic activity. CDK1 phosphorylates AJUBA at Ser119 and Ser175 during the G2/M phase of the cell cycle promoting proliferation and tumorigenesis^101^. Besides these, other downstream oncoprotein substrates of CDK1 (Supplementary Table 3) participate in multiple signaling pathways mediating tumor progression.

Several CDK1 substrates are oncogenic transcription factors. For example, forkhead box M1B (FOXM1B) transcriptional activity requires binding of either S or M phase CDK/cyclin complexes to mediate efficient CDK1 phosphorylation of the FoxM1B Thr596 residue, which is essential for recruitment of CREB binding protein coactivator proteins^102^. Phosphorylation of islet-1 (ISL1) at Ser269 by CDK1 increases its transcriptional activity and promotes cell proliferation in gastric cancer^103^. Mammalian target of rapamycin (mTOR)-directed eukaryotic translation initiation factor 4E-binding protein 1 (4E-BP1) phosphorylation promotes cap-dependent translation and tumorigenesis. CDK1-directed phosphorylation of 4E-BP1 may yield a gain of function activity, distinct from translational regulation, which may be important in tumorigenesis and mitotic centrosome function^104^. The activating transcription factors (ATFs) belong to the activator protein 1 (AP-1) family of transcription factors^105^. Phosphorylation of ATF7 by CDK1 at Thr51 or Thr53 in M phase is required for G2/M progression^106^. Additionally, RUNX family transcription factor 1 (RUNX1)^107^, RUNX2^108^, retinoid X receptor alpha (RXRA)^109^, CCAAT enhancer binding protein alpha (CEBPA)^110^, transcription factor CP2 like 1 (TFCP2L1)^111^, and octamer-binding transcription factor 4 (OCT4)^112,113^ are also oncogenic transcription factors mediated by CDK1 (Supplementary Table 3).

Apart from these CDK1 substrates, BCL2 apoptosis regulator (BCL2), BCL2 apoptosis regulator like 1 (BCL2L1), and dynamin 1 Like (DRP1) are phosphorylated by CDK1, mediating mitochondrial fusion and apoptosis in human cancer cells^114–121^. F-box protein 28 (FBXO28) and carboxypeptidase D (CPD) are phosphorylated by CDK1 increasing ubiquitylation promoting tumorigenesis. FBXO28 ubiquitin ligases act as one of the master regulators of cellular homeostasis by targeting key proteins for ubiquitylation. FBXO28 activity and stability are regulated during the cell cycle by CDK1/2 phosphorylation, which is required for its efficient ubiquitylation of MYC and downstream enhancement of the MYC pathway. CDK1-mediates activation of the FBXO28 ubiquitin ligase promotes MYC-driven transcription and tumorigenesis and predicts poor survival in breast cancer^122^. A GATA family transcription factor, GATA-binding protein 2 (GATA2), participates in cell growth and differentiation of various cells. GATA2 contains CPD, a consensus motif for ubiquitylation that includes Thr176. CDK1 phosphorylates CPD at Thr176, which increases GATA2 expression levels^123^. Moreover, several additional molecules also demonstrate ontogenetic function mediated by CDK1 (Supplementary Table 3).

### CDK1 tumor suppressor substrates

The tumor suppressor p53, an important CDK1 substrate, plays critical roles in a diversity of physiologic functions by increasing genomic stability, inhibiting cell transformation, and initiating apoptosis when DNA damage repair is defective^124^. Cyclin B1/CDK1-mediated Ser315 phosphorylation in p53-wild-type tumor cells may provide insights for improving the efficacy of anti-cancer therapy^125^. Moreover, the tumor protein p73 transcription factor is a member of the p53 family and participates in developmental processes and the DNA damage response. CDK1-dependent Thr86 phosphorylation represses the ability of p73 to induce endogenous p21 expression^126^. The forkhead box O (FOXO) transcription factor FOXO1 functions as a tumor suppressor by mediating apoptosis, cell cycle arrest, and oxidative detoxification. CDK1 may contribute to tumorigenesis by promoting cell proliferation and survival through phosphorylation and inhibition of FOXO1^127,128^. Additionally, tumor suppressors caspase 8 (CASP8) and caspase 9 (CASP9) are phosphorylated by CDK1 facilitating apoptosis in cancer cells^129,130^. Some other CDK1 substrates act as tumor suppressors, including discs large MAGUK scaffold protein 1 (DLG1)^131^, F-box protein 5 (EMI1)^132^, sequestosome 1 (P62)^133^, EPH receptor A2 (EPHA2)^134,135^, and vestigial like family member 4 (VGLL4)^136^. They influence tumor progression through multiple signaling pathways, including the APC, Ras/MAPK, and Hippo pathways. Besides these tumor suppressors, receptor-associated protein 80 (RAP80), inhibitor of growth family member 1 (ING1), and EMAP Like 2 (EML2) are also phosphorylated by CDK1 mediating DNA damage, cell proliferation, and migration^137,138^ (Supplementary Table 3).

### CDK1 cell cycle substrates

The cell cycle consists of the mitotic (M) phase and interphases, G1, S, and G2. CDK1 functions during the entire cell cycle by phosphorylating its various substrates. CDK1 is the major protein kinase that drives cells into mitosis^139^. CDK1 phosphorylates multiple substrates including aurora kinase activator (BORA)^140^, mixed lineage leukemia-5 (MLL5)^141^, and greatwall (GWL)^142^, which all have a critical role in mitotic entry. CDK1 also acts as a mediator in mitotic exit by phosphorylation of cell division cycle associated 5 (CDCA5)^143^, and centromere protein A (CENPA)^144^. M phase consists of four basic phases including prophase, metaphase, anaphase, and telophase. CDK1 phosphorylates non-SMC condensin II complex subunit D3 (CAPD3) at Thr1415, which is required for timely chromosome condensation during prophase^145^. Checkpoint kinase 2 (CHK2) is an essential protein kinase governing DNA damage and replication stress checkpoints. CDK1 phosphorylates CHK2 kinase in metaphase, influencing cellular morphogenesis^146^. The spindle and kinetochore associated complex subunit 3 (SKA3) protein complex is required for accurate chromosome segregation during mitosis^147^. SKA3 is phosphorylated by CDK1 in mitosis to promote the onset of anaphase^148^. CDK1 phosphorylates 4E-BP1 at Ser83, which accumulates at centrosomes during prophase, peaks at metaphase, and decreases through telophase^104^. Besides these, CDK1 also phosphorylates several other substrates during M phase by mediating multiple functions including spindle assembly, microtubule dynamics, and completion of cytokinesis (Supplementary Table 3). Overall, the substrates of CDK1 are critical in M phase for efficient cell division.

Following M phase, CDK1 substrates also function in the interphases of cell cycle. Fatty acyl-CoA reductase 1 (FAR1) transcription is maximal between mitosis and early G1 phase. Phosphorylation (Ser87) by CDK1 primes FAR1 for ubiquitin-mediated proteolysis^149^. At entry into S phase, CDK1 phosphorylates WRN recQ like helicase (WRN)^150^, cell division cycle 7 (CDC7)^151^, BRCA1 DNA repair associated (BRCA1)^152^, and RAD9 checkpoint clamp component A (RAD9)^153^, influencing DNA replication and checkpoint control. In particular, CDK1-mediated phosphorylation of BRACA1 participates in BRCA1-dependent S phase checkpoint control in response to DNA damage^152^. Telomeric repeat factor 1 (TRF1), a duplex telomeric DNA-binding protein, plays an important role in telomere metabolism. CDK1 phosphorites TRF1, which is recruited to sites of DNA damage to facilitate homologous recombination and checkpoint activation at the S/G2 phase^154^. Additionally, CDK1 phosphorylates ELAV like RNA binding protein 1 (ELAVL1) during G2, thereby helping to retain it in the nucleus hindering its post-transcriptional function and anti-apoptotic influence^155^. Besides these, several other molecules also play important roles in cell cycle progression (Supplementary Table 3).

## CDK1 interactome and related signaling pathways

**Fig. 3:**
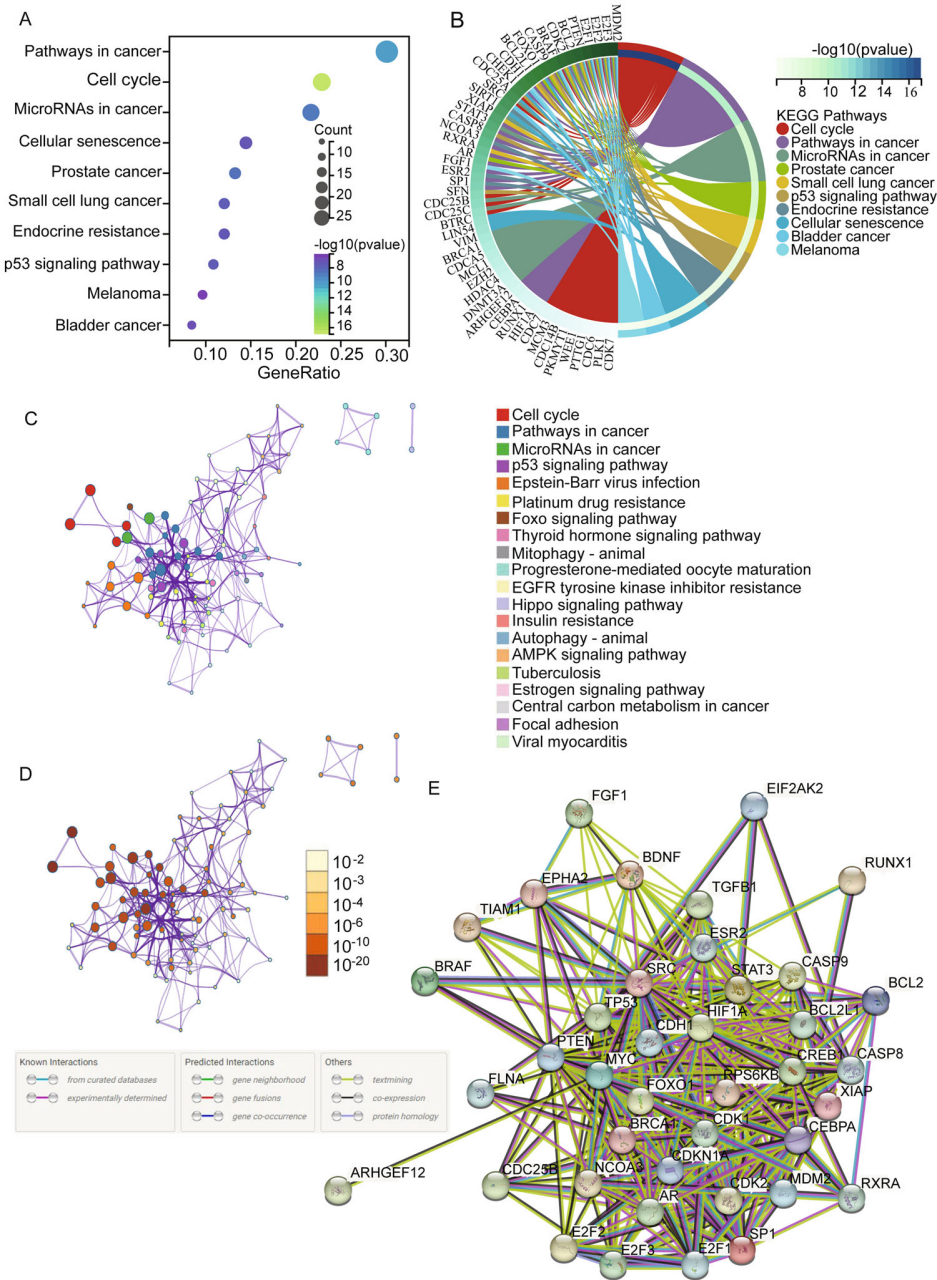
Signaling pathways associated with CDK interacting proteins. **A** Signaling pathways involving CDK1-interacting proteins are identified by KEGG pathway enrichment analyses. Pathways in cancer, cell cycle, and microRNAs and ten top pathways are shown by bubble chart. An FDR of <0.05 was considered statistically significant. **B** KEGG pathway enrichment analyses cluster plot showing a chord dendrogram of the clustering of the expression spectrum of the proteins involve in ten top pathways. **C** The network is visualized by using Cytoscape with “force-directed” layout and with edge bundled for clarity. One term from each cluster is selected to have its description shown as the label. **D** The same enrichment network has its nodes colored by *p* value, as shown in the legend. The darker the color, the more statistically significant is the node (see legend for *p* value ranges). **E** The interacting network of CDK1 and its interacting proteins in Pathways in cancer are demonstrated by using the STRING (https://string-db.org/).

CDK1 participates in tumorigenesis by interacting with many proteins (Supplementary Tables 2 and 3) that have functions in multiple signal pathways. We performed Kyoto Encyclopedia of Genes and Genomes (KEGG) pathway enrichment analyses of potential signaling pathways associated with CDK1 interacting proteins. We used the KEGG rest API (https://www.kegg.jp/kegg/rest/keggapi.html) to obtain the latest gene annotations of the KEGG pathway. The enrichment analysis was performed using the R software package, clusterProfiler (v3.14.3), to obtain results of gene set enrichment. An FDR of <0.05 was considered statistically significant. The results indicate that CDK1 integrating proteins are involved in signaling pathways in cancer, cell cycle, and microRNAs. Ten top pathways (Fig. 3A, B) were identified. Using Metascape, we then selected a subset of representative terms and converted them into a network layout^156^. More specifically, each term is represented by a circle node, where its size is proportional to the number of input genes falling under that term, and its color represents its cluster identity (i.e., nodes of the same color belong to the same cluster). Terms with a similarity score > 0.3 are linked by an edge and the thickness of the edge represents the similarity score. The network is visualized with Cytoscape (v3.1.2) with “force-directed” layout and with edge bundled for clarity. One term from each cluster is selected to have its term description shown as a label (Fig. 3C). The same enrichment network has its nodes colored by the *p* value, as shown in the legend. The darker the color, the more statistically significant the node is (see legend for *p* value ranges, Fig. 3D). Based on the metanalysis results, signal pathways in cancer, cell cycle, and microRNAs in cancer are the top 3 pathways associated with CDK1 interacting proteins. Using the STRING database, we then obtained the interacting network of CDK1 and its interacting proteins in pathways in cancer (Fig. 3E). Collectively, CDK1 is clearly involved in multiple cancer-related pathways, suggesting the significance of CDK1 in various cancer processes.

## Targeting CDK1 provides a potential strategy for attenuating cancer development

This review thus far has examined the critical role of CDK1 in cancer. The accumulated findings demonstrate that CDK1 could be a potential target for cancer prevention and therapy. In recent years, several small molecules with anticancer activity that target CDK1 and other CDKs have been identified in preclinical and clinical studies focusing on multiple cancer types. The effects of various CDK1 associated CKIs in cancer are summarized in Supplementary Tables 4 and 5.

### Targeting CDK1 in preclinical studies

RO-3306^157–159^ and CGP-74514A^160,161^ are specific CDK1 inhibitors that effectively suppress the growth of cancer cells and patient derived xenografts (PDX). Additionally, the pan-CDK inhibitors have shown anticancer activity in preclinical studies (Supplementary Table 4).

### Targeting CDK1 in clinical studies

CDKs are attractive targets against cancer and CDK inhibitors have been studied since the 1990s. Also, various clinical trials have investigated the use of CDK inhibitors in order to improve treatment of patients with virous cancer types (Supplementary Table 5). Some of the more notable inhibitors are discussed below.

#### BEY1107

BEY1107 (avotaciclib) is an orally active CDK1 inhibitor. A phase 1/2 clinical trial has assessed the maximum tolerated dose, safety, and efficacy of BEY1107. It is proposed to be used as a monotherapy and in combination with gemcitabine in patients with locally advanced or metastatic pancreatic cancer^162^.

#### Flavopiridol

Flavopiridol (alvocidib) is a pan-CDK inhibitor that suppresses CDK1, CDK2, CDK4, CDK6, CDK7, and CDK9 with IC_50s_ of 30, 170, 100, 60, 300, and 10 nM, respectively. Several clinical trials have been conducted for the treatment of leukemia^163^, multiple myeloma^164^, sarcoma, gastrointestinal stromal tumor, and other solid tumors. Flavopiridol has received “orphan drug” designation from the FDA for LAML^165^. Previous preclinical studies suggested that flavopiridol can inhibit cancer development^166,167^. Unfortunately, it showed less efficacy in human clinical studies. In particular, flavopiridol at this dose and schedule does not have single-agent activity in patients with colorectal cancer. Trials that evaluate flavopiridol in combination with active cytotoxic drugs should help to define the role of this novel agent in colorectal cancer^168^. Additionally, flavopiridol also exhibited certain side effects in the clinical trial. Flavopiridol as a single agent given by bolus and then infusion caused significant diarrhea, cytopenias, and transaminase elevation, but only achieved marginal responses in relapsed myeloma^164^. To decrease the side effects, the combination of flavopiridol with other anticancer drugs might be an effective way to enhance its efficacy^169^.

#### Roniciclib

Roniciclib (BAY1000394) is an orally bioavailable pan-cyclin dependent kinase (CDK) inhibitor, with IC_50s_ of 5–25 nM for CDK1, CDK2, CDK3, CDK4, CDK7, and CDK9. Roniciclib has been used in several clinical trials of various neoplasms and lung cancer. Based on a Phase 1 dose-escalation study of roniciclib in advanced malignancies, Roniciclib demonstrated an acceptable safety profile and moderate disease control rate in 3 days on/4 days off schedule^170^. Roniciclib co-administered with chemotherapy in patients with extensive-disease small-cell lung cancer (ED-SCLC) demonstrated tolerability, acceptable pharmacokinetics, and promising efficacy. Unfortunately, an observed safety signal in a related phase 2 study resulted in discontinuation of the present study and termination of further development of roniciclib^171,172^.

#### P276-00

P276-00 (Riviciclib) is a potent CDK inhibitor and suppresses CDK1, CDK4, CDK9 activity with IC_50s_ of 79, 63, and 20 nM, respectively. A phase 1 study was designed to determine the maximum tolerated dose, toxicity profile, pharmacokinetics, and anti-cancer activity of P276–00 given intravenously to patients with advanced refractory neoplasms^13,173^. Additional clinical studies evaluated efficacy of P276-00 in subjects with advanced malignant melanoma positive for cyclin D1 expression, advanced triple negative breast cancer, and advanced head and neck cancer^13,174^. Notably, a Phase 2, single-arm, open-label, multicenter study evaluated the efficacy and safety of P276-00 in patients with relapsed or refractory mantle cell lymphoma. Of the 13 patients, 11 experienced disease progression, 1 patient was withdrawn because of an adverse event, and 1 patient died. Given the results observed in the present study, if evaluation of CDK inhibition in MCL continues, it should be considered earlier in the disease course or as a part of combination strategies for relapsed or refractory disease^175^. These results suggest the anticancer efficacy of P276-00; however, further investigations are still needed to confirm the anticancer effects and safety.

#### Dinaciclib

Dinaciclib (SCH727965) is a broad spectrum and competitive inhibitor of CDKs. It can inhibit CDK1, CDK2, CDK5, and CDK9 with IC_50s_ of 3, 1, 1, and 4 nM, respectively. Dinaciclib has been used in several clinical trials to treat multiple cancer types such as pancreatic cancer, non-small-cell lung cancer, neoplasms, leukemia, breast cancer, myeloma, lymphoma and melanoma. Dinaciclib has demonstrated inhibitory effects in several clinical studies and showed significant clinical activity against relapsed and refractory chronic lymphocytic leukemia. Positive responses occurred in 28 (54%) of patients with a median progression-free survival of 481 days^176^. Another study demonstrated single agent activity of dinaciclib against relapsed myeloma^177^. The same study also showed that dinaciclib treatment demonstrated antitumor activity in 2 of 7 patients with estrogen receptor-positive and human epidermal growth factor receptor 2-negative metastatic breast cancer (1 confirmed and 1 unconfirmed partial response), as well as acceptable safety and tolerability^178^. All these results suggested that dinaciclib is a promising CDK1-associated inhibitor for clinical treatment of cancer.

#### AT7519

AT7519 (AT7519M) is a potent inhibitor of CDKs, with IC_50s_ of 210, 47, 100, 13, 170, and 10 nM for CDK1, CDK2, CDK4 to CDK6, and CDK9, respectively. AT7519 shows encouraging anticancer activity against multiple cancer cell lines and tumor xenografts^179,180^. AT7519 has also been evaluated in several clinical trials, including lymphoma and unspecified adult solid tumors, multiple myeloma, and leukemia. A phase 1 study of AT7519 was conducted to evaluate the safety and tolerability. The preliminary anticancer activity was observed with AT7519 at 27.0 mg/m^2^^181^. Additionally, promising preliminary clinical activity was observed when AT7519 was combined with the HSP90 inhibitor onalespib^182^. Collectively, AT7519 is also another promising CDK1-associated inhibitor for cancer treatment in the clinic.

#### Other CDK1 inhibitors

Seliciclib (Roscovitine), AG-024322, PHA-793887, R547, RGB-286638, AZD-5438, and Indirubin (Couroupitine B) exhibited potential for clinical application (Supplementary Table 5). However, assessment of the safety and antitumor activity is still needed in future studies.

## Limitation and potential of targeting CDK1

CDK1-associated inhibitors might replace traditional endocrine therapies in many situations. However, adverse side effects^183,184^ and less efficacy^185–187^ are still limitations for clinical application. Because CDKs play important roles in normal cellular processes, targeting them can lead to unintended consequences, such as toxicity and other adverse effects. Comprehensively understanding the mechanisms by which CDKs contribute to cancer and normal cell functions is crucial to balance the potential benefits of CDK inhibitors with their risks and toxicities. Designing CDK inhibitors that selectively target cancer cells while minimizing toxicity to normal cells requires intricate knowledge of CDK regulation. To address the limitations of CDK inhibitors, combination treatment with other anti-cancer agents might be best use in cancer treatment. Highlighting the distinct regulatory mechanisms of CDK1 activity in different cancer types can enhance precision oncology and enable successful combinatorial treatment with CDK1 inhibitors^188–190^. For instance, CDK1 inhibition can be a potential therapy for MYC-dependent breast cancer^188^. In the clinical application, the toxicities are manageable and clinical activity was observed when flavopiridol in combination with paclitaxel in patients with esophagus, lung, and prostate cancer^169^. The pan-CDK1 inhibitor dinaciclib in combination with rituximab, an anti-CD20 monoclonal antibody, was well tolerated and revealed encouraging clinical activity in relapsed/refractory chronic lymphocytic leukemia patients^191^. In addition, promising preliminary clinical activity has been observed in a Phase 1 study of the HSP90 inhibitor onalespib in combination with AT7519, a pan-CDK inhibitor, in patients with advanced solid tumors^182^. Furthermore, several clinical trials of CDK1 associated inhibitors combining with other anti-cancer agents are ongoing to evaluate the combination treatment in cancer (NCT03579836; NCT03484520; NCT01434316; NCT01676753). These clinical studies will provide more information for the combination of CDK1 associated inhibitors with other anti-cancer agents for cancer treatment.

Besides side effects, several clinical trials indicated that CDK1-associated inhibitors failed to demonstrate sufficient efficacy in cancer patients^185–187^. The preclinical data suggest that the lack of efficacy of CDK1 associated inhibitors might be associated with poor pharmacokinetics of the drugs^192,193^. In addition, clinical trials performed on some cancer patients failed to respond to treatment because of low expression levels of CDK1. Another possible reason for the lack of efficacy could be related to advanced stage of tumor progression enabling more resistance to therapy in general. To improve these issues, screening of patients with high CDK1 expression is important for recruiting patients. Additionally, combination therapy should also be an effective approach to enhance the efficacy of CDK1-associated inhibitors in clinical trials.

## Summary

**Fig. 4:**
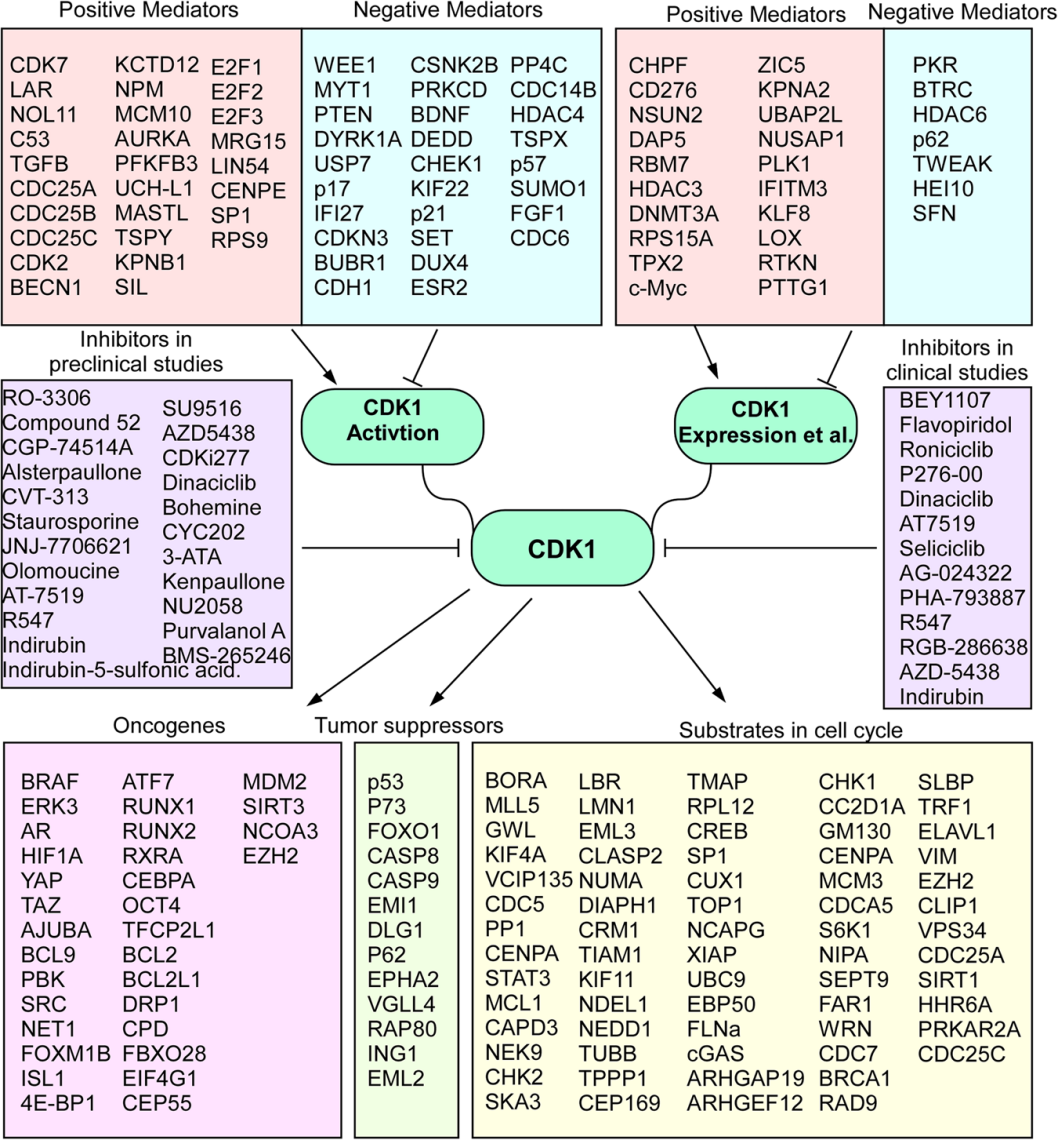
Schematic diagram illustrating the CDK1-associated mediators in cancer. CDK1 expression is regulated at either transcriptional or post-transcriptional levels and CDK1 activity is tightly controlled by numerous molecules. Once activated, CDK1 interacts with and phosphorylates a wide variety of proteins serving as oncogenes, tumor suppressors, or substrates in cell cycle. Selective CKIs and pan-CDK1 have been developed and studied in preclinical or clinical evaluation.

In this review, we focused on the role the CDK1 in cancer and examined the potential application of targeting CDK1 for cancer treatment. We demonstrated the expression level and associated survival rate of CDKs in multiple cancer types. The results suggest that CDK1 is a promising target protein in various cancers. We also examined proteins that interact and mediate CDK1 or are mediated by CDK1. Our analysis demonstrated that CDK1-associated proteins play a critical role in multiple cancer signaling pathways. These results provide evidence of clinical benefits of CKIs. A series of preclinical studies have shown that CKIs mediate various cancer cell processes including proliferation, apoptosis, invasion, and metastasis. Importantly, several preclinical animal studies and clinical studies demonstrated the efficacy of CKIs in cancer treatment. These results are summarized in Fig. 4 and suggest potential opportunities for targeting CDK1 as a cancer treatment.

Overall, this review summarized the function and mechanism of CDK1 in cancer. Targeting CDK1 might provide opportunities for cancer prevention and therapy. The combined CDK1 associated inhibitors with other anticancer agents might improve the chemotherapeutic benefits and improve clinical outcome in cancer development. Future studies are required to determine these issues^194–336^.

## Supplementary information

Supplementary Figure and Tables
