# Supplementary material for: Targeting CDK1 in cancer: mechanisms and implications
Source: NPJ Precis Oncol. 2023 Jun 13;7:58. doi: 10.1038/s41698-023-00407-7 (PMC10264400; doi:10.1038/s41698-023-00407-7)
Supplement: Supplementary file 1 — Supplementary Figure and Tables [file 41698_2023_407_MOESM1_ESM.pdf]

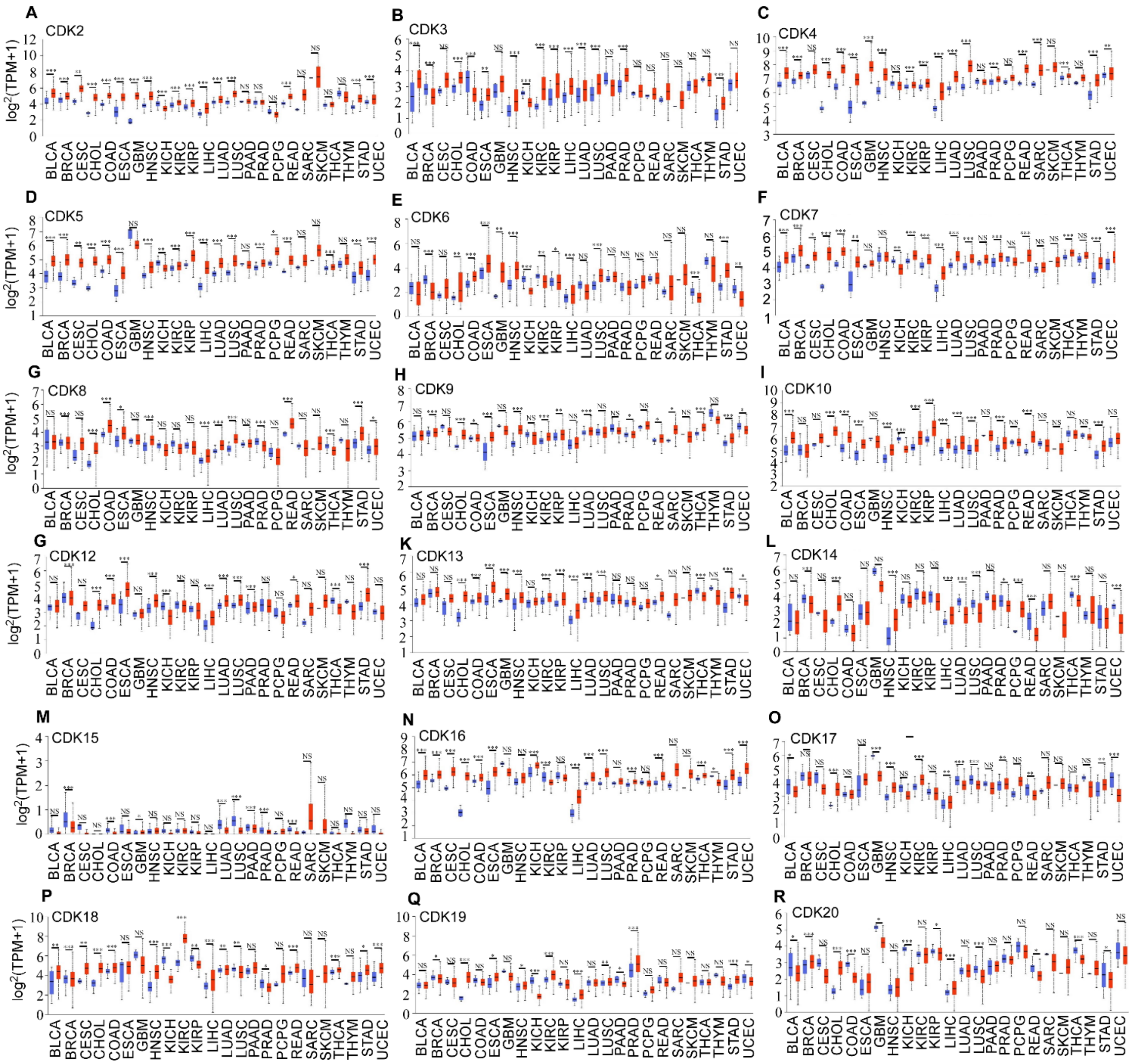

**Supplementary Fig. 1. Expression levels of CDKs in various cancers.** Comparison of the expression of CDKs between tumor (red) and normal (blue) tissues. For the boxplots, the center line of the box indicates the median. The upper boundary of the box represents the upper quantile, while the bottom boundary of the box represents the lower quartile. The top and bottom ends of the whiskers indicate the maximum and minimum values, respectively. (* p  < 0.05, ** p < 0.01, *** p < 0.001, NS: no significant difference).

**Supplementary Table 1. Expression and overall survival rate of patients expressing various CDKs in diverse cancers**

| **Cancer type** | **Acronym** | **Specific CDK(s) overexpressed ($p < 0.05$)** | **Specific CDKs affecting overall survival rate ($p < 0.05$)** |
|---|---|---|---|
| adrenocortical carcinoma | ACC | no data | CDK1, CDK2, CDK3, CDK6, CDK10, CDK17 |
| acute myeloid leukemia | LAML | no data | CDK14, CDK18 |
| bladder urothelial carcinoma | BLCA | CDK1, CDK2, CDK3, CDK4, CDK5, CDK7, CDK10, CDK16, CDK17, CDK18, CDK20 | CDK2, CDK6, CDK10, CDK15, CDK17 |
| brain lower grade glioma | LGG | no data | CDK1, CDK,2, CDK3, CDK6, CDK7, CDK8, CDK12, CDK13, CDK16, CDK20 |
| breast invasive carcinoma | BRCA | CDK1, CDK2, CDK3, CDK4, CDK5, CDK6, CDK7, CDK8, CDK9, CDK12, CDK14, CDK15, CDK16, CDK18, CDK19, CDK20 | CDK8 |
| cervical squamous cell carcinoma | CESC | CDK1, CDK2, CDK5, CDK7, CDK13, CDK16, CDK18, | - |
| cholangiocarcinoma | CHOL | CDK2, CDK3, CDK4, CDK5, CDK6, CDK7, CDK8, CDK9, CDK10, CDK12, | CDK15 |

| | | | |
|---|---|---|---|
| | | CDK13, CDK14, CDK16, CDK17, CDK18, CDK19, CDK20 | |
| colon adenocarcinoma | COAD | CDK1, CDK2, CDK3, CDK4, CDK5, CDK6, CDK7, CDK8, CDK9, CDK10, CDK12, CDK13, CDK15, CDK16, CDK18, CDK20 | CDK10 |
| esophageal carcinoma | ESCA | CDK1, CDK2, CDK3, CDK4, CDK5, CDK6, CDK7, CDK8, CDK9, CDK10, CDK12, CDK13, CDK16, CDK19, | - |
| glioblastoma multiforme | GBM | CDK1, CDK2, CDK4, CDK6, CDK13, CDK15, CDK17, CDK20 | CDK5, CDK7 |
| head and neck squamous cell carcinoma | HNSC | CDK1, CDK2, CDK3, CDK4, CDK5, CDK6, CDK8, CDK9, CDK10, CDK12, CDK14, CDK17, CDK18, CDK19, | CDK3, CDK19 |
| kidney chromophobe | KICH | CDK2, CDK3, CDK5, CDK6, CDK7, CDK10, CDK12, CDK13, CDK16, CDK18, CDK19, CDK20 | CDK1, CDK2, CDK4, CDK5, CDK7, CDK8, CDK12 |
| kidney renal clear cell carcinoma | KIRC | CDK1, CDK2, CDK3, CDK4, CDK5, CDK6, CDK7, CDK9, CDK10, CDK13, CDK16, CDK17, CDK18, CDK19, | CDK1, CDK3, CDK4, CDK**5,** CDK9, CDK10, CDK12 CDK14, CDK15 CDK17, CDK19, CDK20 |

| | | | |
|---|---|---|---|
| kidney renal papillary cell carcinoma | KIRP | CDK1, CDK2, CDK3, CDK4, CDK5, CDK6, CDK7, CDK9, CDK10, CDK13, CDK18, CDK20 | CDK1, CDK2, CDK3, CDK4, CDK7, CDK12 CDK18, CDK19 |
| liver hepatocellular carcinoma | LIHC | CDK1, CDK2, CDK3, CDK4, CDK5, CDK6, CDK7, CDK8, CDK10, CDK12, CDK13, CDK14, CDK16, CDK17, CDK18, CDK19, CDK20 | CDK1, CDK2, CDK4, CDK5, CDK7, CDK8 CDK13, CDK16, CDK19, CDK20 |
| lung adenocarcinoma | LUAD | CDK1, CDK2, CDK3, CDK4, CDK5, CDK7, CDK8, CDK9, CDK10, CDK12, CDK13, CDK14, CDK15, CDK16, CDK17, CDK18, | CDK1, CDK6 |
| lung squamous cell carcinoma | LUSC | CDK1, CDK2, CDK3, CDK4, CDK5, CDK6, CDK7, CDK8, CDK10, CDK12, CDK14, CDK15, CDK16, CDK17, CDK18, CDK19, CDK20 | - |
| lymphoid neoplasm diffuse large B-cell lymphoma | DLBC | no data | - |
| mesothelioma | MESO | no data | CDK1, CDK2, CDK4, CDK6, CDK8, CDK16, CDK19 |
| ovarian serous cystadenocarcinoma | OV | no data | CDK8, CDK14, CDK15, CDK19 |

| | | | |
|---|---|---|---|
| pancreatic adenocarcinoma | PAAD | CDK12, CDK15, CDK19, | CDK1, CDK2 CDK3, CDK6, CDK7, CDK10 |
| prostate adenocarcinoma | PRAD | CDK1, CDK3, CDK4, CDK4, CDK5, CDK7, CDK8, CDK9, CDK10, CDK14, CDK15, CDK16, CDK17, CDK18, CDK19, | CDK7 |
| pheochromocytoma and paraganglioma | PCPG | CDK5, CDK13, CDK14, | CDK1, CDK3, CDK16, CDK19 |
| rectum adenocarcinoma | READ | CDK1, CDK2, CDK4, CDK5, CDK7, CDK8, CDK9, CDK10, CDK12, CDK14, CDK15, CDK16, CDK17, CDK18, CDK20 | CDK12 |
| sarcoma | SARC | CDK4, CDK9, | CDK6, CDK9, CDK14, CDK18 |
| skin cutaneous melanoma | SKCM | CDK13, | CDK1, CDK2, CDK4 |
| testicular germ cell tumors | TGCT | no data | - |
| thyroid carcinoma | THCA | CDK4, CDK5, CDK6, CDK7, CDK8, CDK9, CDK12, CDK14, CDK16, CDK18, CDK20 | CDK15, CDK20 |
| thymoma | THYM | CDK16, | CDK2, CDK7, CDK14, CDK18 |
| stomach adenocarcinoma | STAD | CDK1, CDK2, CDK3, CDK4, CDK6, CDK7, CDK8, CDK9, CDK10, CDK12, | no data |

| | | | |
|---|---|---|---|
| | | CDK13, CDK16, CDK17, CDK18, CDK19, CDK20 | |
| UCEC and uveal melanoma | UVM | no data | CDK2, CDK5, CDK10, CDK15 |
| uterine corpus endometrial carcinoma | UCEC | CDK1, CDK2, CDK4, CDK6, CDK7, CDK8, CDK9, CDK13, CDK14, CDK16, CDK17, CDK18, CDK19, | CDK6, CDK14 CDK16, CDK19, CDK20 |
| uterine carcinosarcoma | UCS | no data | CDK7 |

| **Supplementary Table 2. Upstream mediators of CDK1** | | | |
|---|---|---|---|
| **Positive mediators of CDK1** | | | |
| **Mediator** | **Function** | **Mechanism** | **Ref** |
| CDK7 | Activates CDK1 | Increases phosphorylation of CDK1 at Thr161 | [27] |
| LAR | Activates CDK1 | LAR protein tyrosine phosphatase mediates focal adhesion by enhancing phosphorylation of CDK1 at Thr161 | [28] |
| NOL11 | Activates CDK1 | Depletion of NOL11 delays entry into the M phase owing to increased inhibitory phosphorylation of CDK1 at Tyr15 | [29] |
| C53 | Activates CDK1 | C53 promotes CDK1 activation and mitotic entry in both unperturbed cell cycle progression and DNA damage response | [30] |
| CDC25A | Activates CDK1 | CDC25A mediates the activity of CDK1–2/cyclin A and CDK1/cyclin B in G2 phase | [27 32] |
| CDC25B | Activates CDK1 | CDC25B activates human CDK1 kinase by dephosphorylating both Thr14 and Tyr15 | [33] |
| CDC25C | Activates CDK1 | CDC25C positively mediates activation of CDK1/cyclin B | [34,35] |
| CDK2 | Activates CDK1 | Cyclin A/CDK2 complexes are key mediators of CDK1 activation in human cells | [36] |

| | | | |
|---|---|---|---|
| BECN1 | Activates CDK1 | BECN1 promotes radiation-induced G2/M arrest through mediation of CDK1 activity: a potential role for autophagy at the G2/M checkpoint | [40] |
| KCTD12 | Activates CDK1 | KCTD12 binds to CDC25B and activates CDK1 and Aurora kinase A to facilitate the G2/M transition and promote tumorigenesis | [41] |
| NPM | Activates CDK1 | Phosphorylation at Ser10 and Ser70 of NPM plays a role in mediating CDK1 kinase activity during the G2/M transition | [42] |
| MCM10 | Activates CDK1 | MCM10 activates CDK1 in G2 phase | [43] |
| AURKA | Activates CDK1 | Aurora kinase A is required for initial activation of CDK1 at centrosomes | [39] [44,194] |
| PFKFB3 | Activates CDK1 | PFKFB3 promotes cell cycle progression and suppresses apoptosis through CDK1-mediated phosphorylation of p27 | [45] |
| UCH-L1 | Activates CDK1 | UCH-L1 physically interacts with CDK1 enhancing its kinase activity and promoting cell proliferation | [46] |
| MASTL | Activates CDK1 | MASTL enhances cyclin B1/CDK1-dependent mitotic phosphorylation events, directing mitotic entry, anaphase and cytokinesis in human cells | [47] |
| TSPY | Activates CDK1 | TSPY enhances the cyclin B1-CDK1 phosphorylation activity in modulating cell-cycle progression at the G2/M stage | [48] |
| KPNB1 | Activates CDK1 | KPNB1 promotes CDK1/cyclin B1 translocation from the cytoplasm to the nucleus | [49] |
| SIL | Activates CDK1 | Knockdown of SIL in cancer cells *in vitro* delays entrance into mitosis and decreases activation of the CDK1/cyclin B complex | [50] |

| | | | |
|---|---|---|---|
| E2F1-3 | Promote CDK1 transcription | E2F1, E2F2, and E2F3 bind to the CDK1 promoter promoting its transcription | [51] |
| MRG15 | Promotes CDK1 transcription | MRG15 acts in a HAT complex involving Tip60 to modify chromatin through the acetylation of histone H4 at the CDK1 promoter to activate transcription | [52] |
| LIN54 | Promotes CDK1 transcription | LIN54 is an essential core subunit of the DREAM/LINC complex that binds to the CDK1 promoter and promotes CDK transcription | [53] |
| CENPE | Promotes CDK1 transcription | Overexpression of CENPE significantly increases CDK1 promoter activity | [54] |
| SP1 | Promotes CDK1 transcription | Overexpression of SP1 significantly decreases G2/M arrest in cervical cancer cells, which is related to upregulation of CDK1 expression | [56] |
| RPS9 | Promotes CDK1 transcription | Knockdown of RPS9 inhibits the growth of human colon cancer cells at the G2/M phase by downregulating CDK1 expression at the promoter level | [57] |
| CHPF | Enhances CDK1 expression | CHPF promotes development of malignant melanoma through mediation of CDK1 | [58] |
| CD276 | Enhances CDK1 expression | CD276 maintains proliferation and increases differentiation by enhancing CDK1 expression in endothelial progenitor cells | [59] |
| NSUN2 | Enhances CDK1 translation | NSUN2 methylates *CDK1* mRNA enhancing CDK1 translation | [60,61] |

| | | | |
|---|---|---|---|
| DAP5 | Enhances CDK1 translation | The translation initiation factor DAP5 is a mediator of cell survival during mitosis by enhancing CDK1 translation | [62] |
| RBM7 | Stabilizes *CDK1* mRNA | Oncogenic action of the exosome cofactor RBM7 by stabilization of *CDK1* mRNA in breast cancer | [63] |
| HDAC3 | Stabilizes CDK1 expression | HDAC3 controls G2/M phase progression mainly through post translational stabilization of CDK1 | [64] |
| DNMT3A | Enhances CDK1 expression | DNMT3A mutation-induced CDK1 overexpression promotes leukemogenesis | [66] |
| RPS15A | Enhances CDK1 expression | RPS15A overexpression induces CDK1 expression | [195] |
| TPX2 | Enhances CDK1 expression | Knocking down TPX2 inhibits CDK1 expression and represses phosphorylation of the ERK/GSK3β/SNAIL signaling pathway, thereby inhibiting the tumor epithelial-mesenchymal transition | [196] |
| MYC | Enhances CDK1 expression | MYC regulates the CDK1/cyclin B1 dependent-G2/M cell cycle progression | [197] |
| ZIC5 | Enhances CDK1 expression | ZIC5 promotes colorectal cancer cell proliferation and cell cycle progression by enhancing CDK1 signaling | [198] |
| KPNA2 | Enhances CDK1 expression | KPNA2 promotes tumor cell proliferation by increasing the expression of CDK1 | [199] |

| | | | |
|---|---|---|---|
| UBAP2L | Enhances CDK1 expression | UBAP2L knockdown decreases CDK1 expression and plays an oncogenic role in HCC | 200 |
| NUSAP1 | Enhances CDK1 expression | NUSAP1 enhances proliferation, migration and invasion in breast cancer cells by increasing CDK1 expression | 201 |
| PLK1 | Enhances CDK1 expression | Activation of PLK1 enhanced CDK1 expression in cells | 202,203 |
| IFITM3 | Enhances CDK1 expression | IFITM3 activates the STAT3/CDK1 pathway | 204 |
| KLF8 | Enhances CDK1 expression | KLF8 knockdown induces cell phase arrest decreasing the expression of CDK1 | 205 |
| LOX | Enhances CDK1 expression | LOX knockdown reduces CDK1 expression in cancer cell mitosis | 206 |
| RTKN | Enhances CDK1 expression | Knockdown of RTKN affects proliferation and metastasis of colon cancer by reducing expression of CDK1 | 207 |
| PTTG1 | Enhances CDK1 expression | Overexpression of PTTG1 causes upregulation of CDK1 expression resulting in enhanced proliferation | 208 |
| **Negative mediators of CDK1** | | | |
| **Mediator** | **Function** | **Mechanism** | **Ref** |
| WEE1 | Inactivates CDK1 | WEE1 kinase phosphorylates the CDK1-cyclin B complex and suppresses the CDK1-cyclin B kinase activity | 67-69 |

| | | | |
|---|---|---|---|
| MYT1 | Inactivates CDK1 | The human MYT1 kinase preferentially phosphorylates CDK1 (Thr14) and localizes to the endoplasmic reticulum and Golgi complex | [70] [71] [72] |
| PTEN | Inhibits CDK1 activation | PTEN decreases phosphorylation of CDK1 (Tyr15), resulting in decreased G2/M cell cycle arrest | [74] |
| DYRK1A | Inactivates CDK1 | Inhibition of DYRK1A results in massive cyclin B accumulation and saturation of CDK1 activity | [75] |
| USP7 | Inactivates CDK1 | USP7 limits CDK1 activity throughout the cell cycle | [209] |
| p17 | Inactivates CDK1 | p17 suppresses CDK1 action and disrupts vimentin phosphorylation, causing G2/M cell cycle arrest | [210] |
| IFI27 | Inactivates CDK1 | IFI27 knockdown increases CDK1 phosphorylation (Tyr15) involved in cell proliferation. | [211] |
| CDKN3 | Inactivates CDK1 | CDKN3 controls mitosis by inhibiting CDK1 activation by dephosphorylation of CDK1 (Thr161) | [212] |
| BUBR1 | Inhibits CDK1 activation | Deletion of BURR1 activates cyclin B/CDK1 kinase, leading to premature mitotic entry | [213] |
| CDH1 | Inhibits CDK1 activation | UV-induced degradation of CDH1 results in accumulation of cyclin B1 and thus increased CDK1 activity | [214] |
| CSNK2B | Inhibits CDK1 activation | Knockdown of CSNK2B causes stabilization of WEE1 and increases phosphorylation of CDK1 (Tyr15) | [215] |

| | | | |
|---|---|---|---|
| PRKCD | Inactivates CDK1 | PRKCD induces phosphorylation of CDK1 (Tyr15), a critical event in the G2/M checkpoint | [216] |
| BDNF | Inhibits both CDK1 expression & activity | Inhibition of both CDK1 expression and activity through a BDNF-dependent mechanism contributes to the maintenance of tetraploid RGCs in a G2-like state | [217] |
| DEDD | Inactivates CDK1 | DEDD is an inhibitor of mitotic CDK1/cyclin B1 | [79,80] |
| CHEK1 | Inhibits CDK1 activation | CHEK1 prevents entry into mitosis by inhibiting CDK1 activity | [76,77] |
| KIF22 | Inactivates CDK1 | Inhibition of KIF22 increases CDC25C expression and CDK1 activity, resulting in delayed mitotic exit | [78] |
| p21 | Inactivates CDK1 | Loss of p21CIP1 unleashes CDK1 activity | [218] [219] |
| SET | Inactivates CDK1 | SET inhibits cyclin B/CDK1 activity | [220,221] |
| DUX4 | Inactivates CDK1 | DUX4 functions as a direct inhibitor of CDK1, and decreases colon cancer cell proliferation | [222] |
| ESR2 | Inactivates CDK1 | Unliganded ESR2 causes a G2 cell cycle arrest by inactivating CDK1 | [223] |
| PP4C | Inactivates CDK1 | Loss of PP4C leads to an unscheduled activation of CDK1 in interphase | [224] |
| CDC14B | Inhibits CDK1 activation | CDC14B counteracts CDK1 action and causes mitotic arrest in mouse pre-implantation embryo models | [225] |
| HDAC4 | Inhibits CDK1 activation | HDAC4 inhibits the CDK1 activity and the progression of proliferating HEK293T and HT22 cells through the cell cycle. | [226] |

| | | | |
|---|---|---|---|
| TSPX | Inhibits CDK1 activation | TSPX represses the cyclin B1/CDK1 phosphorylation activity | [48] |
| p57 | Inhibits CDK1 activation | p57 is required to trigger endoreduplication by inhibiting CDK1 | [227] |
| SUMO1 | Inhibits CDK1 activation | Inhibits CDK1 activity by SUMOylation | [228] |
| FGF1 | Inhibits CDK1 activation | FGF1 inhibits the cyclin B1/CDK1 kinase activity to induce a transient G2 arrest | [229] |
| CDC6 | Inhibits CDK1 activation | Phosphorylation of CDC6 promotes the interaction of CDC6 and CDK1, leading to attenuated CDK1 activity | [230] |
| PKR | Induces CDK1 degradation | PKR mediates Tyr4 phosphorylation facilitating CDK1 ubiquitination and triggering CDK1 downregulation | [82] |
| BTRC | Induces CDK1 degradation | CDK1 accumulation in patients' tumors shows a negative correlation with BTRC and a positive correlation with the degree of tumor malignancy | [83] |
| HDAC6; p62 | Induces CDK1 degradation | p62/SQSTM1/HDAC6-dependent autophagy mediates CDK1 degradation in human breast cancer | [87] |
| TWEAK | Induces CDK1 degradation | TWEAK induces CDK1 phosphorylation and degradation, resulting in the arrest of cell growth at the G2/M phase | [231] |
| HEI10 | Inhibits CDK1 expression | HEI10 negatively mediates cell invasion by inhibiting cyclin B/CDK1 expression | [89] |

| | | | |
|---|---|---|---|
| SFN | Inhibits CDK1 nuclear translocation | SFN is required to sequester CDK1/cyclin B1 complexes in the cytoplasm and prevent mitotic catastrophes | [90] |

| **Supplementary Table 3. Downstream substrates of CDK1** | | | |
|---|---|---|---|
| **CDK1 tumor promotor substrates** | | | |
| **Target substrate** | **Phosphorylation site** | **Biological function** | **Ref** |
| BRAF | Ser144 | CDK1/cyclin B directly phosphorylates BRAF at Ser144, which is required for mitotic activation of BRAF and subsequent activation of the MAPK cascade | [91] |
| ERK3 | Thr698 | CDK1 phosphorylates ERK3 at Thr698, which operates in a cell-cycle-dependent manner | [92] |
| AR | Ser81, Ser 515 | CDK1 phosphorylates the AR at Ser81 and leads to AR-mediated transactivation in prostate cancer; phosphorylation of AR at Ser515 by CDK1 may be an independent prognostic marker | [93-95] |
| HIF1A | Ser668 | CDK1 stabilizes HIF1A through direct phosphorylation of Ser668 to promote tumor growth | [98] |
| YAP | Thr119, Ser289, Ser367 | CDK1 phosphorylates YAP, promoting mitotic defects and cell motility | [99] |
| TAZ | Ser90, Ser105, Thr326, Thr346 | CDK1 phosphorylation of TAZ in mitosis inhibits its oncogenic activity | [100] |
| AJUBA | Ser119, Ser175 | Adaptor protein AJUBA is phosphorylated by CDK1 at Ser119 and Ser175 during the G2/M phase of the cell cycle, which promotes cell proliferation and tumorigenesis. | [101] |

| | | | |
|---|---|---|---|
| BCL9 | Thr172 | CDK1-mediated BCL9 phosphorylation in controlling mitotic Wnt signaling to promote cell division and growth | [232] |
| PBK | Thr24, Ser32, Se59 | CDK1-mediated mitotic phosphorylation of PBK is involved in cytokinesis and inhibits its oncogenic activity | [233] |
| SRC | Thr34, Thr46, Ser72 | SRC phosphorylation by CDK1 has been demonstrated to increase SRC kinase activity | [234] |
| NET1 | Ser131, Thr146 | CDK1 phosphorylation mediates the activity of NET1 towards RhoA during mitosis. | [235] |
| FOXM1B | Thr596, Ser251 | CDK1 phosphorylates FOXM1B, which is essential for recruitment of CREB binding protein coactivator proteins. | [102,236] |
| ISL1 | Ser269 | Phosphorylation of ISL1 at Ser269 by CDK1 increases its transcriptional activity and promotes cell proliferation in gastric cancer | [103] |
| 4E-BP1 | Ser83 | CDK1 phosphorylation of mRNA translation regulator 4E-BP1 at Ser83, which contributes to cell transformation | [104] |
| ATF7 | Thr51, Thr53 | CDK1-mediated phosphorylation of human ATF7 at Thr51 and Thr53 promotes cell-cycle progression into M phase | [106] |
| RUNX1 | Ser48, Ser303, Ser424 | Phosphorylation of RUNX1 by CDK1 reduces direct interaction with HDAC1 and HDAC3 | [107] |
| RUNX2 | Ser451 | Mediates G2 and M phases and promotes tumor angiogenesis | [108] [237] |

| | | | |
|---|---|---|---|
| RXRA | Ser265 | CDK1 phosphorylates RXRA by activating c-Jun $NH_2$-terminal kinases | [109] |
| CEBPA | Ser21 | CDK1 phosphorylates CEBPA on Ser21, which inhibits its differentiation-inducing function | [110] |
| OCT4 | Ser229 | CDK1 interplays with OCT4 to repress differentiation of embryonic stem cells into trophectoderm | [113] |
| TFCP2L1 | Thr177 | TFCP2L1 is phosphorylated at Thr177 by CDK1, which affects embryonic stem cell cycle progression, pluripotency and differentiation | [111] |
| BCL2 | Ser70 | Mediates BCL2 phosphorylation and link the coupling of mitotic arrest and apoptosis | [114,115,117,118] |
| BCL2L1 | Ser62 | Mediates BCL2L1 phosphorylation and links coupling mitotic arrest and apoptosis | [114,116,118] |
| DRP1 | Ser585; Ser616 | DRP1 is specifically phosphorylated in mitosis by CDK1/cyclin B on Ser585; DRP1 mediates mitochondrial fusion and apoptosis in human cancer cells | [119-121] |
| CPD | Thr176 | The CPD kinase can mediate CDK phosphorylation at Thr176 | [123] |
| FBXO28 | Ser344 | CDK1 mediates activation of the FBXO28 ubiquitin ligase promoting MYC-driven transcription and tumorigenesis in breast cancer | [122] |
| EIF4G1 | Ser1232 | Phosphorylation of EIF4G1 (Ser1232) in the mitotic translation initiation shift | [238] |

| | | | |
|---|---|---|---|
| CEP55 | Ser425, Ser428 | CEP55 is triggered by CDK1-dependent phosphorylation at Ser425 and Ser428, which is required for its recruitment to midbody and cytokinesis | [239] |
| MDM2 | Thr216 | MDM2 is phosphorylated by cyclin A/CDK2 or cyclin A/CDK1 at Thr216, which is most prevalent at the onset of S phase | [240] |
| SIRT3 | Thr150, Ser159 | SIRT3 enzymatic activity is further enhanced through of Thr150/Ser159 phosphorylation by cyclin B1-CDK1, which is also induced by radiation and relocated to mitochondria together with SIRT3 | [241] |
| NCOA3 | Ser728, Ser867 | Phosphorylation of NCOA3 at mitosis is mediated by CDK1 | [242] |
| EZH2 | Thr487 | CDK1-dependent phosphorylation of EZH2 suppresses methylation of H3K27 and promotes osteogenic differentiation of human mesenchymal stem cells | [243] |
| **CDK1 tumor suppressor substrates** | | | |
| **Target substrate** | **Phosphorylation site** | **Biological function** | **Ref** |
| p53 | Ser315 | Anti-apoptotic function of mitochondrial p53 mediated by cyclin B1/CDK1 Ser315 phosphorylation in p53-wildtype tumor cells | [125] |
| p73 | Thr86 | p73 proteins are targets of CDK complexes and phosphorylation on Thr86 by CDK1 mediates p73 functions | [126] |

| | | | |
|---|---|---|---|
| FOXO1 | Ser249 | CDK1 contributes to tumorigenesis by promoting cell proliferation and survival through phosphorylation and inhibition of FOXO1 | [127,128] |
| CASP8 | Ser387 | Inhibits apoptosis during mitosis and contributes to tumorigenesis | [129,244] |
| CASP9 | Thr125 | Cyclin/CDK1 phosphorylates CASP9 on Thr125 and protects mitotic cells from apoptosis | [130] |
| EMI1 | Ser145, Ser149 | EMI1 is phosphorylated by CDK1 providing a delay for APC activation | [132] |
| DLG1 | Ser158, Ser442 | DLG1 is phosphorylated by CDK1 on Ser158 and Ser442, which affect the nuclear localization of DLG1. | [131] |
| P62 | Thr269, Ser272 | Phosphorylation of p62 by CDK1 controls the timely transit of cells through mitosis and tumor cell proliferation | [133] |
| EPHA2 | Ser897 | EPHA2 phosphorylation on Ser897 by the CDK1/MEK/ERK/RSK pathway mediates M-phase progression by maintaining cortical rigidity. | [245] |
| VGLL4 | Ser58, Ser155, Thr159, Ser280 | CDK1 mediates mitotic phosphorylation of the transcriptional co-repressor VGLL4 and inhibits its tumor-suppressing activity | [136] |
| RAP80 | Ser677 | CDK1 mediates phosphorylation of RAP80 at Ser677 and modulates DNA damage-induced G2/M checkpoint and cell survival | [137] |
| ING1 | Ser126 | CDK1 phosphorylates Ser126 of ING1, which plays a key role in mediating the expression of cyclin B1 and proliferation of melanoma cells | [246] |

| | | | |
|---|---|---|---|
| EML2 | Thr84 | CDK1 phosphorylates EML2, modulating cell proliferation and migration. | [138] |
| ***CDK1 cell cycle substrates*** | | | |
| **Target substrate** | **Phosphorylation site** | **Biological function** | **Ref** |
| BORA | Thr12, Thr15, Ser25, Ser41, Thr52, Ser112, Ser137, Ser183, Thr243, Ser252, Ser271, Ser274, Ser278, Thr287, Ser375, Thr381 | Phosphorylation of the N terminus of BORA for PLK1 activation and mitotic entry | [140] |
| MLL5 | Thr912 | Phosphorylation of MLL5 by CDK1 affects its cellular distribution and is required for mitotic entry | [141] |
| GWL | T193, T206 | CDK1 phosphorylates GWL during mitotic entry | [142] |
| KIF4A | Ser1186 | CDK1-dependent KIF4A phosphorylation at Ser1186 is a trigger for chromosomal organization during early mitosis | [247] |
| VCIP135 | Ser130 | In early mitosis, phosphorylation of VCIP135 by CDK1 at a single residue, Ser130, is sufficient to inactivate the enzyme and inhibit p97/p47-mediated Golgi membrane fusion | [248] |

| | | | |
|---|---|---|---|
| CDC5 | Thr242 | Mitotic exit function of Polo-like kinase CDC5 is dependent on sequential activation by CDK1 | [143] |
| PP1 | Thr320 | CDK1 inhibits PP1-mediated dephosphorylation at mitotic exit | [249] |
| CENPA | Ser68 | Eliminates the binding of CENPA to the assembly factor HJURP, thus preventing the premature loading of CENPA to the centromere prior to mitotic exit | [144] |
| STAT3 | Ser727 | Phosphorylation of STAT3 (Ser727) by CDK1 is critical for nocodazole-induced mitotic arrest | [250] |
| MCL1 | Thr92 | Phosphorylation of MCL1 by CDK1-cyclin B1 during mitotic arrest | [251] |
| CAPD3 | Thr1415 | Thr1415 of the CAP-D3 subunit is a CDK1 phosphorylation site, which is required for timely chromosome condensation during prophase | [145] |
| NEK9 | Thr210 | CDK1 is necessary for NEK9 activation for centrosome separation during mitosis. | [252] |
| CHK2 | Ser774 | CDK1 phosphorylates CHK2 kinase in metaphase, influencing cellular morphogenesis | [146] |
| SKA3 | T358, T360 | SKA3 is phosphorylated by CDK1 in mitosis to promote anaphase onset | [148] |
| LBR | Ser71, Ser86 | CDK1 activity prevents premature nuclear envelope assembly and phosphorylation of the inner nuclear membrane protein LBR by CDK1 contributes to the temporal control | [253] |

| | | | |
|---|---|---|---|
| LMN1 | Ser22, Ser392 | The nuclear envelope is a dynamic structure that completely disassembles in response to CDK1 activity in mitosis | [254] |
| EML3 | Thr881 | CDK1 phosphorylates EML3 regulating mitotic spindle assembly and the kinetochore-microtubules connection | [255] |
| CLASP2 | Ser1234 | CDK1 mediates CLASP2 stabilizing kinetochore-microtubule attachments | [256] |
| NUMA | Thr2055 | CDK1-mediated phosphorylation at T2055 negatively modulates NUMA cortical localization mediating mitotic progression with proper spindle function. | [257] |
| DIAPH1 | Ser629, Ser640, Ser665 | CDK1-mediated DIAPH1 phosphorylation maintains metaphase cortical tension and inactivates the spindle assembly checkpoint at anaphase | [258] |
| CRM1 | Ser391 | Phosphorylation of CRM1 by CDK1/cyclin B promotes Ran-dependent mitotic spindle assembly | [259] |
| TIAM1 | Ser1466 | S1466 of TIAM1 is a novel CDK1 site for which phosphorylation is required for the mitotic function of TIAM1 to promote mitotic spindle formation | [260] |
| KIF11 | Thr926 | CDK1 phosphorylates KIF11 at Thr926 during mitosis, which is crucial for the mitotic function of KIF11 | [261] |

| | | | |
|---|---|---|---|
| NDEL1 | Thr219 | Silencing of CDK1 clearly suppresses the phosphorylation of NDEL1 at Thr219 mediating microtubule organization | 224 |
| NEDD1 | Thr550 | Promotes the interaction of NEDD1 with the γTuRC component γ-tubulin during mitosis | 262 |
| TUBB | Ser172 | Mediation of microtubule dynamics during mitosis. | 263 |
| TPPP1 | Thr14, Ser18, Ser45, Ser160 | CDK1 phosphorylation of TPPP1 inhibits its modulation of cell cycle to increase cell proliferation | 264 |
| CEP169 | Ser451 | Mediates microtubule dynamics of mitotic spindle | 265 |
| TMAP | Thr622 | CDK1/cyclin B1-mediated phosphorylation of TMAP contributes to proper regulation of microtubule dynamics and establishment of functional bipolar spindles during mitosis | 266 |
| RPL12 | Ser38 | Phosphorylation of the ribosomal protein RPL12 by CDK1 affects translation during mitosis | 267 |
| CREB | Ser270, Ser271 | CDK1-dependent phosphorylation of CREB at Ser270 and Ser271 facilitates its dissociation from chromatin during mitosis by reducing its intrinsic DNA binding potential | 268 |
| SP1 | Ser720, Thr723, Thr737 | CDK1-mediated SP1 phosphorylation facilitates cell cycle progression during mitosis | 269 |

| | | | |
|---|---|---|---|
| CUX1 | Ser1237, Ser1270 | Hyperphosphorylation of CUX1 by CDK1/cyclin B inhibits its DNA binding activity in mitosis | [270] |
| TOP1 | Ser112, Ser394 | TOP1 is phosphorylated during mitosis by CDK1, which enhances DNA relaxation activity | [271] |
| NCAPG | Thr308, Thr332 | Phosphorylation of NCAPG is required for its chromosomal DNA localization during mitosis | [272] |
| XIAP | Ser40 | Phosphorylation of XIAP by CDK1-cyclin-B1 controls mitotic cell death | [273] |
| UBC9 | Ser71 | Phosphorylation of UBC9 by CDK1 enhances SUMOylation activity in cell cycle mediation | [274] |
| EBP50 | Ser279, Ser301 | CDK1/cyclin B-mediated EBP50 phosphorylation plays a role in the modulation of various cell functions by affecting actin cytoskeleton reorganization | [275] |
| FLNa | Ser1436 | CDK1/cyclin B1 phosphorylates Ser1436 of FLNa mediating actin remodeling | [276] |
| cGAS | Ser305 (human)<br>Ser291 (mouse) | DNA sensor cGAS is post-translationally modulated by cell cycle-dependent enzymes to ensure its proper activation for host defense of cytosolic DNA in interphase and is inert to self-DNA in mitosis | [277] |
| ARHGAP19 | Thr404, Thr476 | Phosphorylation of ARHGAP19 by CDK1 mediates its subcellular localization and function during mitosis | [278] |

| | | | |
|---|---|---|---|
| ARHGEF12 | Ser190, Ser1176 | CDK1 phosphorylates ARHGEF12 at Ser190 and Ser1176 during mitosis | [279] |
| CHK1 | Ser286, Ser301 | CHK1 is phosphorylated at Ser286 and Ser301 by CDK1 during mitosis | [76] |
| CC2D1A | Ser208 | CDK1 is a kinase of CC2D1A during mitosis and its phosphorylation of CC2D1A mediates mitotic function | [280] |
| GM130 | Ser25 | GM130 is phosphorylated in early mitosis on Ser25 by CDK1/cyclin B. | [281] |
| CENPA | Ser68 | Dynamic phosphorylation of CENPA at Ser68 by CDK1 orchestrates its cell-cycle-dependent deposition at centromeres | [144] |
| MCM3 | Ser112 | CDK1-dependent phosphorylation of Ser112 triggers the assembly of MCM3 with the remaining MCM subunits and subsequent chromatin loading of MCMs | [282] |
| CDCA5 | Ser21, Thr48, Ser75, Ser79, Ser83, Thr111, Thr115, Thr159, Ser181, Ser209 | phosphorylation of CDCA5 by CDK1 influences sister chromatid cohesion | [283,284] |
| S6K1 | Ser371, Ser411, Thr421, Ser424 | CDK1 triggers inactivation of S6K1 in mitosis, serving to spare energy for costly mitotic processes at the expense of ribosomal protein synthesis | [285] |

| | | | |
|---|---|---|---|
| NIPA | Ser395 | CDK1/cyclin B1 amplifies phosphorylation of NIPA, which may contribute to the regulation of its own abundance in early mitosis | [286] |
| SEPT9 | Thr24 | Mitotic regulation of SEPT9 by CDK1 is important for the completion of cytokinesis | [287] |
| FAR1 | Ser87 | Phosphorylation by CDK1 primes FAR1 for ubiquitin-mediated proteolysis | [149] |
| WRN | Ser1133 | CDK1 phosphorylates WRN at collapsed replication forks | [150] |
| CDC7 | Ser16, Ser302, Thr376, Thr472, Thr503 | CDK1-mediated phosphorylation of CDC7 suppresses DNA re-replication | [151] |
| BRCA1 | Ser1497, Ser1189, Ser1191 | CDK1 participates in BRCA1-dependent S phase checkpoint control in response to DNA damage | [152] |
| RAD9 | Ser11 | Dynamics of RAD9 chromatin binding and checkpoint function are mediated by CDK1 activity | [153] |
| SLBP | Thr61 | Phosphorylation of Thr61 by cyclin A/CDK1 triggers degradation of stem-loop binding protein at the end of S phase | [288] |
| TRF1 | Thr371 | CDK1 phosphorylates TRF1, which is recruited to sites of DNA damage to facilitate homologous recombination and checkpoint activation | [154] |

| | | | |
|---|---|---|---|
| ELAVL1 | Ser202 | CDK1 phosphorylates ELAVL1 during G2, thereby helping to retain it in the nucleus hindering its post-transcriptional function and anti-apoptotic influence | [155] |
| VIM | Ser55 | Mediates proliferation of mammalian cell lines | [289] |
| EZH2 | Thr350 | Link between the cell-cycle machinery and epigenetic gene silencing | [290] |
| CLIP1 | Thr287 | CDK1-mediated phosphorylation of CLIP1is essential for the normal function of this protein during cell cycle progression | [291] |
| VPS34 | Thr159 | Negative regulation of VPS34 by CDK1 mediates cell-cycle progression | [292] |
| CDC25A | Ser17, Ser115<br>Ser115, Ser320 | Mitotic stabilization of CDC25A reflects its phosphorylation on Ser17 and Ser115 by CDK1/cyclin B, which is required to uncouple CDC25A from its ubiquitin-proteasome-mediated turnover | [293,294] |
| SIRT1 | Thr530, Ser540 | Mutation of these two residues phosphorylated by CDK1/cyclin B disturbs normal cell cycle progression and fails to rescue proliferation defects in SIRT1-deficient cells | [295,296] |
| HHR6A | Ser120 | CDK1-mediated phosphorylation of HHR6A, which is an important regulatory event in the control of cell cycle progression | [297] |
| PRKAR2A | Thr54 | Thr54 phosphorylation of PRKAR2A by CDK1 might serve to mediate PRKACA during cell cycle | [298] |

| | | | |
|---|---|---|---|
| CDC25C | Ser214 | The cell cycle control protein CDC25C is present, and phosphorylated on Ser214 by CDK1 in the transition from germinal vesicle to metaphase II in human oocyte meiosis | [299] |

| Supplementary Table 4. CDK1 inhibitors in preclinical studies | | | | | |
|---|---|---|---|---|---|
| **Compound name** | **Structure** | **Target ($IC_{50}$)** | **Cell base potency ($IC_{50}$ or effective inhibitory dose)** | **Animal model (type and dose and route of administration)** | **Ref** |
| RO-3306 | 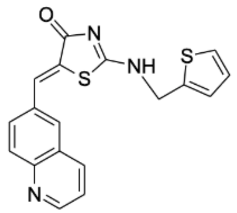 | CDK1 (35 nM) | *$IC_{50}$:*<br>WAC2 (1.8 µM)<br>SHEP (1.3 µM)<br>NB69 (2.3 µM)<br>SK-N-FI (4 µM)<br>NLF (3.8 µM)<br>RH-41 (> 8 µM)<br>IMR32 (1.6 µM)<br>IMR-32 LV-h-p53 (2.4 µM)<br>NGP (4 µM)<br>HDMB-3 (1.8 µM) | Patient derived xenograft (PDX) tumor model– RO3306 4 mg/kg every 2 days, oral gavage administration | [157-159] |

| | | | | | |
|---|---|---|---|---|---|
| CGP-74514A | 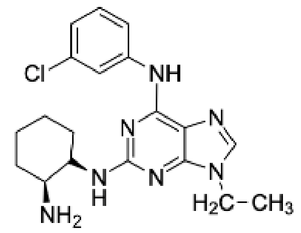 | CDK1 (25 nM) | *$IC_{50}$:*<br>MCF-7 (5 µM)<br>T47D (5 µM)<br>SK-BR-3 (5 µM)<br>NCl-H720 (2.2 µM)<br>NCl-H727 (3.2 µM)<br>BON-1 (1.9 µM)<br>hTERT-RPE1 (13 µM) | NA | [160,161] |
| Alsterpaullone | 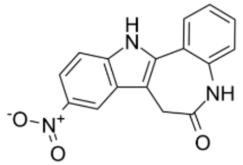 | CDK1 (35 nM)<br>CDK2 (15 nM)<br>CDK5 (40 nM) | *Effective inhibitory dose*<br>D425 (5 µM)<br>D458 (5 µM) | D458 cerebellar xenograft model– alsterpaullone 30 mg/kg, SC, daily for 2 weeks; | [300,301] |
| CVT-313 | 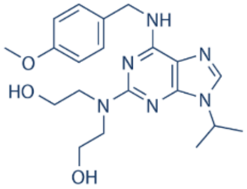 | CDK1 (4.2 µM)<br>CDK2 (500 nM)<br>CDK4 (215 µM) | A2058 (16 µM)<br>Caco-2  (4.5 µM)<br>Capan-1 (10 µM)<br>L1210 (4.2 µM)<br>MCF-7 (6.25 µM)<br>MRC-5 (20 µM)<br>Neonatal (5 µM)<br>RVSMC (1.25 µM) | Rat restenosis model—Rats were injected with CBT-313 (0.75 and 0.25 mg/kg) intraperitoneally | [302,303] |

| | | | | | |
|---|---|---|---|---|---|
| | | | P-388D1 (4 µM)<br>Panc1 (8 µM) | | |
| Indirubin | 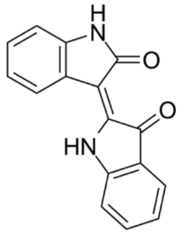 | CDK1 (10 µM)<br>CDK2 (2.2 µM)<br>CDK4 (12 µM)<br>CDK5 (5.5 µM) | *Effective inhibitory dose:*<br>HaCaT (100 nM) | Full-thickness wounds were created on the dorsal skin of BALB/c mice, which were then treated with vaseline containing DMSO (1%) or indirubin (262.26 ng/g vaseline). | [304,305] |
| Indirubin-5-sulfonic acid. | 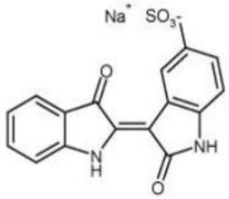 | CDK1 (55 nM)<br>CDK2 (35 nM)<br>CDK4 (300 nM)<br>CDK5 (65 nM) | *Effective inhibitory dose:*<br>HUVEC (25 µM) | Human prostate xenograft mouse model–indirubin solution was intralesional injected at a dose of 10 mg/kg daily | [304] |
| Staurosporine | 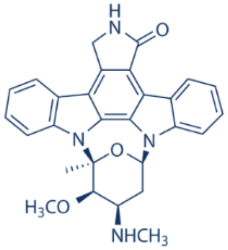 | CDK1 (5 nM)<br>CDK2 (7 nM)<br>CDK4 (<10 µM)<br>CDK5 (4 nM) | *$IC_{50}$:*<br>Primary hOB (11.63 µM)<br>U2OS (6.69 µM)<br>HOS (6 µM)<br>Saos-2 (6.16 µM)<br>T-24 (0.029 µM) | Bladder T-24 xenograft model–athymic nude mice were treated with staurosporine 0.1 mg/kg daily by intraperitoneal injection | [306-308] |

| | | | | | |
|---|---|---|---|---|---|
| | | | HL-60 (0.13 μM)<br>CEC (0.022 μM) | | |
| JNJ-7706621 | 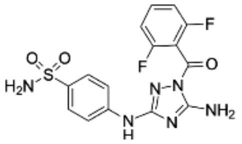 | CDK1 (9 nM)<br>CDK2 (4 nM) | *$IC_{50}$:*<br>WAC2<br>(10.6 μM)<br>SHEP (1 μM)<br>NB69 (2.3 μM)<br>SK-N-FI (4.1 μM)<br>NLF (3.4 μM)<br>RH-41 (2.8 μM)<br>IMR-32 (1.3 μM)<br>IMR-32 LV-h-p53<br>(2.1 μM)<br>NGP (3.1 μM)<br>HDMB-3 (1.6 μM) | A375 melanoma human tumor xenograft model– JNJ-7706621 was administered to mice orally at 100 or 125 mg/kg daily | [158,309] |
| Olomoucine | 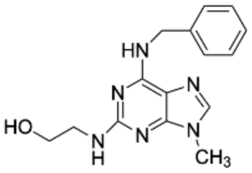 | CDK1 (7 μM)<br>CDK2 (7 μM)<br>CDK5 (3 μM) | *$IC_{50}$:*<br>HL-60 (40 μM)<br>A2780 (30 μM)<br>A2780Cis$^{R}$ (45 μM) | HCT116 colon xenograft model–<br>Olomoucine was administered in a volume | [310,311] |

| | | | | | |
|---|---|---|---|---|---|
| | | | CH1 (48 µM)<br>CH1Cis$^{R}$ (78 µM)<br>CH1Cox$^{R}$ (86 µM)<br>SKOV-3 (> 50 µM)<br>BE (> 50 µM)<br>HT29 (58 µM)<br>Mawi (> 50 µM)<br>Lovo (> 50 µM)<br>SW620 (> 50 µM)<br>HCT116 (52 µM)<br>COLO-205 (41 µM)<br>KM12 (90 µM)<br>SA-OS2 (78 µM)<br>U2-OS (101.5 µM)<br>MCF 7 (64 µM)<br>MB-MDA231 (101.5 µM)<br>A549 (54.5 µM)<br>MOR (66 µM)<br>HX147 (106.5 µM)<br>CORL23 (54 µM) | of 0.1 mL/10 g bodyweight in 50 mmol/L by IV | |

| | | | | | |
|---|---|---|---|---|---|
| | | | GCT (27 µM) | | |
| AT-7519 | 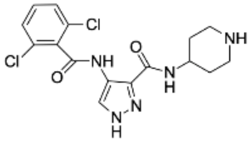 | CDK1 (220 nM)<br>CDK2 (44 nM)<br>CDK4 (67 nM)<br>CDK5 (11 nM) | *$IC_{50}$:*<br>HeLa (0.325 µM)<br>ME-180 (0.599 µM) | Xenograft tumor model–AT7519 was administrated at 15 mg/kg body weight once a day for 5 days for a 2-week duration by IV. | [312,313] |
| R547 | 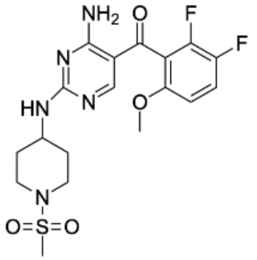 | CDK1 (0.001 µM)<br>CDK2 (0.003 µM)<br>CDK4 (0.001 µM) | *$IC_{50}$:*<br>MDA-MB-468 (0.11 µM)<br>MDA-MB-435 (0.08 µM)<br>MCF-7 (0.06 µM)<br>HCT116 (0.08 µM)<br>SW480 (0.07 µM)<br>RKO (0.05 µM)<br>HT-29 (0.17 µM)<br>HCT15 (0.61 µM)<br>H460a (0.06 µM)<br>C33A (0.32 µM)<br>DU145 (0.08 µM)<br>OSA-CL (0.19 µM) | Xenograft models–R547 was administered at 40, 60 or 80 mg/kg with formulated as a suspension in Klucel LF/Tween 80 or R547 was formulated as a solution in hydroxylpropyl h-cyclodextrin, sodium hydroxide, and water for IV injection at 3.75, 7.5, 15, or 30 mg/kg. | [314] |

| | | | | | |
|---|---|---|---|---|---|
| | | | LOX (0.05 μM)<br>JEKO-1 (0.08 μM)<br>REC-1 (0.09 μM) | | |
| SU9516 | 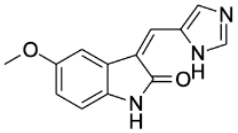 | CDK1 (0.04 μM)<br>CDK2 (0.022 μM) | *$IC_{50}$:*<br>A431 (2.2 μM)<br>H460 (3.6 μM)<br>Colo-205 (2.6 μM)<br>RKO (5.6 μM)<br>SW480 (6.4 μM) | Duchenne muscular dystrophy mouse model–mice were administered 5 mg/kg SU9516 by oral gavage from 3 to 10 weeks of age | [315,316] |
| AZD5438 | 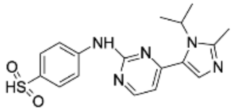 | CDK1 (16 nM)<br>CDK2 (6 nM)<br>CDK9 (20 nM) | *$IC_{50}$:*<br>MCF-7 (0.22 μM)<br>MCF-7Adr (0.31 μM)<br>MDA-MB-23 (0.46 μM)<br>HCT-116 (0.32 μM)<br>HCT-15 (1.13 μM)<br>HT29 (1.05 μM)<br>LoVo (0.63 μM)<br>SW620 (0.58 μM)<br>Colo-205 (0.7 μM)<br>A549 (0.57 μM) | BT474c, CoLo-205, HX147, PC-3, SW620 cell line xenografts–The mice were orally administered AZD5438 at 25, 50, or 100 mg/kg, daily | [317] |

| | | | | | |
|---|---|---|---|---|---|
| | | | H322 (0.4 µM)<br>H460 (0.87 µM)<br>PC-3 (0.2 µM)<br>DU145 (0.42 µM)<br>A2780 (1.26 µM)<br>HeLa (1.1 µM)<br>IM-9 (1 µM)<br>MOLP-8 (1.1 µM)<br>AMO-1 (1 µM)<br>ARH-77 (1.7 µM)<br>KARPAS-620 (0.74 µM)<br>JJN-3 (0.94 µM)<br>L-363 (0.5 µM) | | |
| CDKi277 | 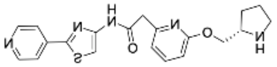 | CDK1 (0.008 µM)<br>CDK2 (0.004 µM)<br>CDK5 (0.005 µM)<br>CDK6 (0.051 µM) | *$IC_{50}$:*<br>Daudi (0.171 µM)<br>HL60 (0.129 µM)<br>K562 (0.438 µM)<br>Jurkat (0.14 µM)<br>HS294 (0.221 µM)<br>A375 (0.12 µM) | Colo205 and PC-3 xenograft models–mice were administered CDKi277 at 25 or 12.5 mg/kg for 4 days on and 1 day off, for three | [318] |

| | | | | | |
|---|---|---|---|---|---|
| | | | MiaPaca2 (0.116 μM)<br>PC-3 (0.251 μM)<br>SaSO2 -Rb (0.32 μM)<br>U2OS+Rb (0.28 μM)<br>Colo205 (0.278 μM)<br>Colo320 (0.241 μM)<br>HCT116 p53$^{+}$ (0.29 μM,<br>HCT116 p53$^{-}$ (0.31 μM)<br>MDA-MB-231 (0.137 μM)<br>MCF-7 p53(+) (0.18 μM)<br>MCF-7 p53(-) (0.14 μM) | consecutive cycles by IP injection | |
| Dinaciclib | 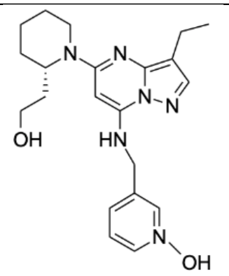 | CDK1 (3 nM)<br>CDK2 (1 nM)<br>CDK5 (1 nM)<br>CDK9 (4 nM) | *Effective inhibitory dose:*<br>BT474 (10 nM)<br>MCF7 (1 nM)<br>T47D (1 nM) | PDX mouse model– Dinaciclib alone was administered by intraperitoneal injection at a dose of 50 mg/kg/day, 5 days a week for total of 4 weeks | [319,320] |

| | | | | | |
|---|---|---|---|---|---|
| Bohemine | 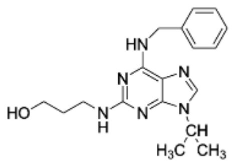 | CDK1 (3.89 µM)<br>CDK2 (4.6 µM)<br>CDK9 (2.7 µM) | *$IC_{50}$:*<br>A2780 (12.5 µM),<br>CH1 (21.5 µM)<br>CH1Cis$^{R}$ (22 µM)<br>CH1Cox$^{R}$ (19 µM)<br>SKOV-3 (81 µM)<br>BE (25 µM)<br>HT29 (27.7 µM)<br>Mawi (28.5 µM)<br>Lovo (25 µM)<br>SW620 (27 µM)<br>HCT116 (17 µM)<br>COLO-205 (21 µM)<br>KM12 (32 µM)<br>SA-OS2 (38 µM)<br>U2-OS (36 µM)<br>MCF 7 (21.5 µM)<br>MB-MDA231 (40 µM)<br>A549 (20 µM)<br>MOR (36 µM) | HCT116 xenograft model–<br>Bohemine were given i.v. in a volume of 0.1 mL/10 g bodyweight in 50 mM HCl/saline at 50 mg/kg | [311,321] |

| | | | | | |
|---|---|---|---|---|---|
| | | | HX147 (38 µM)<br>CORL23 (29.5 µM)<br>GCT (14.5 µM) | | |
| CYC202 | 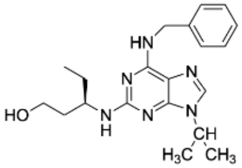 | CDK1 (0.65 µM)<br>CDK2 (0.7 µM)<br>CDK5 (0.16 µM)<br>CDK7 (0.46 µM)<br>CDK9 (0.6 µM) | *$IC_{50}$:*<br>A2780 (4.9 µM)<br>A2780Cis$^{R}$ (8.4 µM)<br>CH1 (7.7 µM)<br>CH1Cis$^{R}$ (9.3 µM)<br>CH1Cox$^{R}$ (7.4 µM)<br>SKOV-3 (31 µM)<br>BE (17.5 µM)<br>HT29 (20.3 µM)<br>Mawi (18 µM)<br>Lovo (20 µM)<br>SW620 (23 µM)<br>HCT116 (6.9 µM)<br>COLO-205 (8.5 µM)<br>KM12 (15 µM)<br>SA-OS2 (16.5 µM)<br>U2-OS (15 µM) | HCT116 tumor xenograft–mice were orally administered CYC202 at 50, 500, or 2,000 mg/kg, daily | [311,322] |

| | | | | | |
|---|---|---|---|---|---|
| | | | MCF 7 (7.8 μM)<br>MB-MDA231 (15 μM)<br>A549 (9.3 μM)<br>MOR (12.5 μM)<br>HX147 (19 μM)<br>CORL23 (10.5 μM)<br>GCT (5.2 μM) | | |
| 3-ATA | 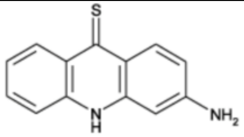 | CDK1<br>(88 μM)<br>CDK2 (>100 μM)<br>CDK4 (3.1 μM) | *Effective inhibitory dose:*<br>cerebellar granule neurons<br>(10 μM) | NA | [323,324] |
| Kenpaullone | 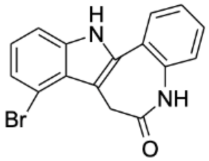 | CDK1 (0.4 μM)<br>CDK2 (0.7 μM)<br>CDK5 (0.9 μM) | *Effective inhibitory dose:*<br>MDA-MB-231 (5 μM) | NA | [12,325] |
| NU2058 | 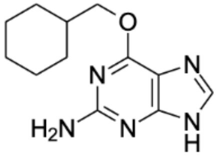 | CDK1 (26 μM)<br>CDK2 (17 μM) | $IC_{50}$:<br>LNCaP (15 μM)<br>LNCaP-cdxR (17 μM)<br>LNCaP-AI (10 μM)<br>PC3 (38 μM) | NA | [326,327] |

| | | | | | |
|---|---|---|---|---|---|
| | | | DU145 (14 μM)<br>CWR22Rv1 (16 μM) | | |
| Purvalanol A | 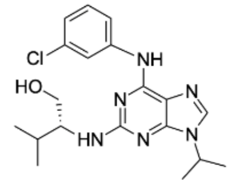 | CDK1 (4 μM)<br>CDK2 (35 μM)<br>CDK4 (850 μM)<br>CDK5 (75 μM) | *Effective inhibitory dose:*<br>MCF-7 (25 μM)<br>MDA-MB-231 (25 μM)<br>HCT 116 (10 μM)<br>HT29 (5 μM)<br>SW480 (5 μM) | NA | 328-332 |
| BMS-265246 | 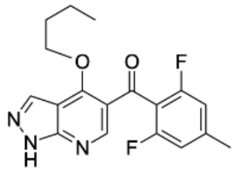 | CDK1 (6 nM)<br>CDK2 (9 nM) | *$IC_{50}$:*<br>Hep-3B (2.84 μM)<br>Hep-G2 (1.73μM) | NA | 333 |

**Supplementary Table 5. CDK inhibitors in clinical development**

| Drug Name | Structure | Major Targets | Phase | Clinical Trial ID | Cancer type | Ref |
|---|---|---|---|---|---|---|
| BEY1107 | 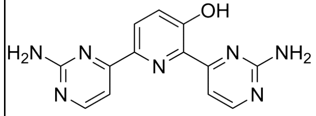 | CDK1 | 1, 2 | NCT03579836 | Locally Advanced or Metastatic Pancreatic Cancer | [162] |
| Flavopiridol | 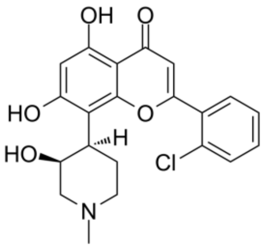 | CDK1/2/4/7/9 | 1, 2 | NCT02520011<br>NCT00112723<br>NCT00005974<br>NCT00098579<br>NCT00007917<br>NCT00324480 | Leukemia, Multiple Myeloma, Sarcoma, Gastrointestinal Stromal Tumor, Lymphoma, Solid Tumors | [163,164] |
| Roniciclib | 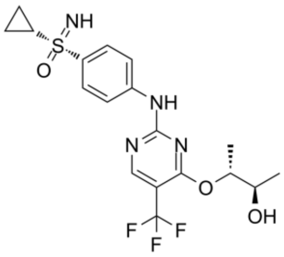 | CDK1/2/3/4/7/9 | 1, 2 | NCT01188252<br>NCT02047890<br>NCT01335256<br>NCT02161419<br>NCT01573338 | Neoplasms, Lung Cancer | [170,171] |

| | | | | | | |
|---|---|---|---|---|---|---|
| P276-00 | 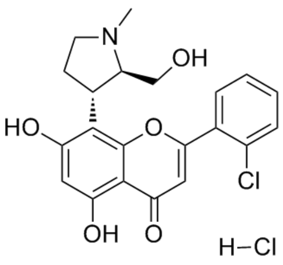 | CDK1/4/9 | 1, 2 | NCT00408018<br>NCT00407498<br>NCT00835419<br>NCT01333137<br>NCT00824343<br>NCT00882063 | Neoplasm,<br>Melanoma,<br>Breast Cancer,<br>Multiple Myeloma,<br>Advanced Head and Neck Cancer,<br>Lymphoma | [173]<br>[334]<br>[13]<br>[174] |
| Dinaciclib | 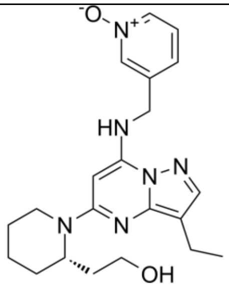 | CDK1/2/5/9 | 1, 2, 3, 4 | NCT01783171<br>NCT00732810<br>NCT03484520<br>NCT01624441<br>NCT01676753<br>NCT01096342<br>NCT02684617<br>NCT01711528<br>NCT01650727<br>NCT00937937<br>NCT01515176<br>NCT01434316<br>NCT01624441 | Pancreatic cancer,<br>Non-Small-Cell Lung cancer,<br>Neoplasms,<br>Leukemia, Breast cancer, Myeloma,<br>Lymphoma,<br>Melanoma | [176-178] |

| | | | | | | |
|---|---|---|---|---|---|---|
| AT7519 | 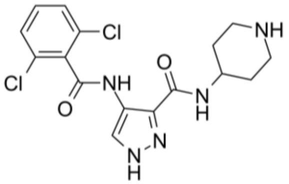 | CDK1/2/4/6/9 | 1, 2 | NCT00390117<br>NCT01652144<br>NCT01627054<br>NCT01183949 | Lymphoma,<br>Unspecified Adult Solid Tumor,<br>Multiple Myeloma,<br>Lymphoma | [181,182,312] |
| Seliciclib | 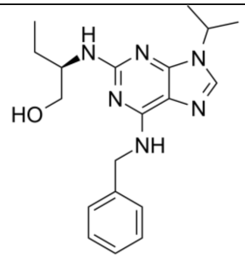 | CDK1/2/5/7 | 1,2 | NCT00999401<br>NCT00372073<br>NCT01333423 | Non-small Cell Lung Cancer, Breast Cancer, Advanced Solid Tumors | [335] |
| AG-024322 | 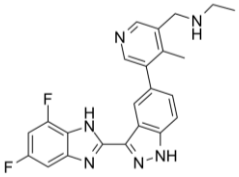 | CDK1/2/4 | 1 | NCT00147485 | Neoplasms,<br>Non-Hodgkin Lymphoma, | [13] |
| PHA-793887 | 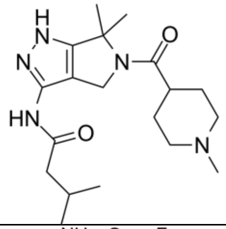 | CDK1/2/4/5 | 1 | NCT00996255 | Advanced/Metastatic Solid Tumors | [334] |
| R547 | 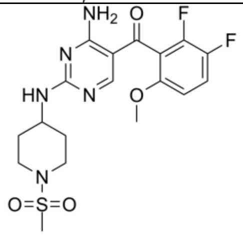 | CDK1/2/4/7 | 1 | NCT00400296 | Neoplasms | [13] |

| | | | | | | |
|---|---|---|---|---|---|---|
| RGB-286638 | 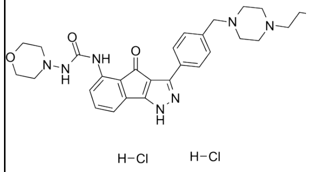 | CDK1/2/3/4/5/9 | 1 | NCT01168882 | Hematological Malignancies | |
| AZD-5438 | 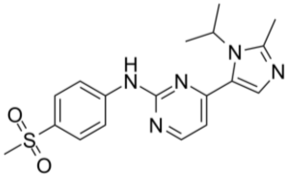 | CDK1/2/9 | 1 | NCT00088790 | Neoplasms | |
| Indirubin | 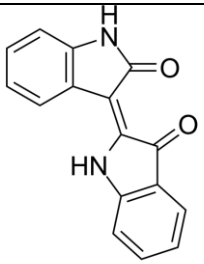 | CDK1/2/4/5 | 4 | NCT02200978 | Childhood Acute Promyelocytic Leukemia | 336 |
